# Loss of tumor-derived SMAD4 enhances primary tumor growth but not metastasis following BMP4 signalling

**DOI:** 10.1186/s12964-024-01559-0

**Published:** 2024-04-30

**Authors:** Lap Hing Chi, Andrew D. Redfern, Suraya Roslan, Ian P. Street, Allan D. Burrows, Robin L. Anderson

**Affiliations:** 1 Olivia Newton-John Cancer Research Institute, 145 Studley Road, Heidelberg, VIC 3084 Australia; 2 School of Cancer Medicine, La Trobe University, Bundoora, VIC Australia; 3 Harry Perkins Institute of Medical Research, University of Western Australia, Perth, WA Australia; 4 Department of Surgery, St. Vincent’s Hospital, Fitzroy, VIC Australia; 5 Children’s Cancer Institute, University of New South Wales, New South Wales, Australia; 6 Department of Clinical Pathology, The University of Melbourne, Parkville, VIC Australia; 7 Sir Peter MacCallum Department of Oncology, The University of Melbourne, Parkville, VIC Australia

**Keywords:** Breast cancer, Metastasis, BMP4, SMAD4, MYO1F, Patient outcomes

## Abstract

**Background:**

Bone morphogenetic protein 4 (BMP4) is a potent inhibitor of breast cancer metastasis. However, a tumor-promoting effect of BMP4 is reported in other tumor types, especially when SMAD4 is inactive.

**Methods:**

To assess the requirement for SMAD4 in BMP4-mediated suppression of metastasis, we knocked down SMAD4 in two different breast tumors and enforced SMAD4 expression in a third line with endogenous SMAD4 deletion. In addition, we assessed the requirement for SMAD4 in tumor cell-specific BMP signalling by expression of a constitutively active BMP receptor. Delineation of genes regulated by BMP4 in the presence or absence of SMAD4 was assessed by RNA sequencing and a BMP4-induced gene, MYO1F was assessed for its role in metastasis. Genes regulated by BMP4 and/or SMAD4 were assessed in a publicly available database of gene expression profiles of breast cancer patients.

**Results:**

In the absence of SMAD4, BMP4 promotes primary tumor growth that is accompanied by increased expression of genes associated with DNA replication, cell cycle, and MYC signalling pathways. Despite increased primary tumor growth, BMP4 suppresses metastasis in the absence of tumor cell expression of SMAD4. Consistent with the anti-metastatic activity of BMP4, enforced signalling through the constitutively active receptor in SMAD4 positive tumors that lacked BMP4 expression still suppressed metastasis, but in the absence of SMAD4, the suppression of metastasis was largely prevented. Thus BMP4 is required for suppression of metastasis regardless of tumor SMAD4 status. The BMP4 upregulated gene, MYO1F, was shown to be a potent suppressor of breast cancer metastasis. Gene signature upregulated by BMP4 in the absence of SMAD4 was associated with poor prognosis in breast cancer patients, whereas gene signature upregulated by BMP4 in the presence of SMAD4 was associated with improved prognosis.

**Conclusions:**

BMP4 expression is required for suppression of metastasis regardless of the SMAD4 status of the tumor cells. Since BMP4 is a secreted protein, we conclude that it can act both in an autocrine manner in SMAD4-expressing tumor cells and in a paracrine manner on stromal cells to suppress metastasis. Deletion of SMAD4 from tumor cells does not prevent BMP4 from suppressing metastasis via a paracrine mechanism.

**Supplementary Information:**

The online version contains supplementary material available at 10.1186/s12964-024-01559-0.

## Background

Breast cancer is the most commonly diagnosed cancer globally, accounting for 2.3 million new cases and over 680,000 deaths each year [1]. Standard therapies frequently become ineffective for suppression of metastatic disease [2]. Recent advances using targeted therapies for metastatic breast cancer [3–5] are prolonging survival, but still only 29% of patients with metastatic breast cancer survive more than five years after diagnosis [6].

We have shown previously that bone morphogenetic protein 4 (BMP4) is lost in more metastatic breast tumor lines and that enforced expression of BMP4 suppresses metastasis [7, 8]. BMP4 is a cytokine in the transforming growth factor β (TGFβ) superfamily, widely known for its involvement in development and bone homeostasis [9]. We have reported a robust anti-metastatic effect of BMP4 signalling in pre-clinical models of breast cancer [7, 8] and highlighted the potential of activating BMP4 signalling as a viable therapeutic approach to treat metastatic breast cancer. This is supported by several publications from some groups [10, 11], whilst contradicted by others [12, 13], necessitating further research to confirm our findings.

The mechanisms by which BMP4 inhibits metastasis have not been fully elucidated. We have reported previously that BMP4 secretion from tumor cells suppresses the activity of myeloid-derived suppressor cells (MDSCs) and may thereby promote anti-tumor immune responses [7] through paracrine signalling from BMP4 expressing tumors. Interestingly, the anti-metastatic effect of BMP4 is retained in immunodeficient mice [8], indicating the involvement of additional immune-independent mechanisms, such as cell cycle arrest [14] or the induction of anoikis [8] in cancer cells.

However, a tumor-promoting role for BMP4 has been reported in other cancer types [15], potentially through the induction of epithelial-to-mesenchymal transition (EMT) [16–18]. These conflicting observations indicate that not all patients with advanced cancer would benefit from a BMP4-activating therapy, highlighting the need for a deeper understanding of the anti-metastatic actions of BMP4.

Discrepancies in the consequences of BMP4 signalling may be explained by the status of the canonical and non-canonical signalling pathways in different cancer types [19]. BMP4 initiates signalling by binding to the type I and type II BMP receptors that in turn phosphorylate transcription factors mothers against decapentaplegic-1, -5 and -8 (SMAD1/5/8). SMAD4, an essential mediator of canonical signalling, is subsequently recruited to pSMAD1/5/8 to form a heteromeric transcriptional factor complex that promotes or represses the expression of target genes [20, 21].

**Fig. 1:**
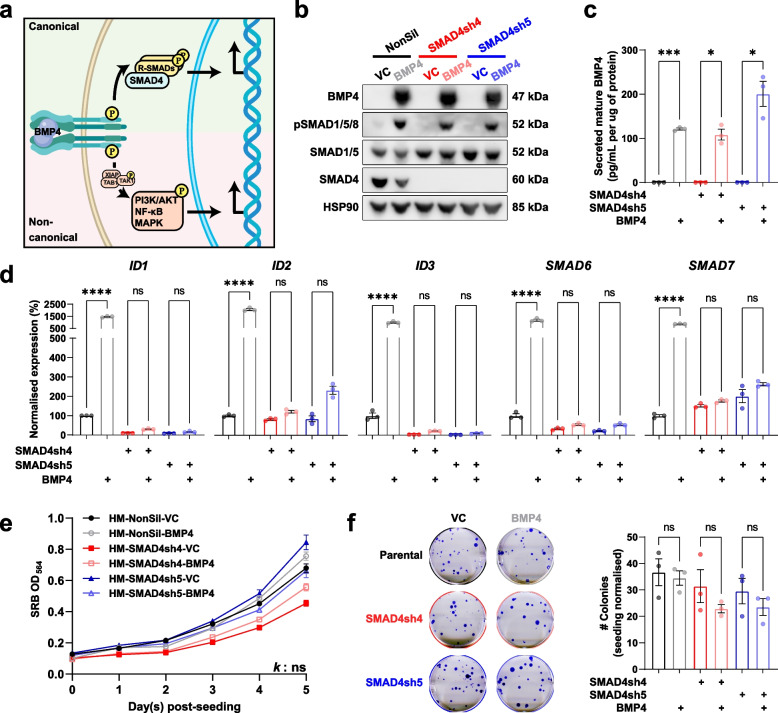
Modification of MDA-MB-231-HM (231-HM) breast cancer cells to investigate canonical and non-canonical BMP4 signalling. **a** Schematic diagram of canonical and non-canonical BMP4 signalling. **b** Western blotting validation of enforced BMP4 expression, SMAD1/5/8 signalling and knockdown of SMAD4 using two different short hairpin RNA constructs in 231-HM cells. **c** Quantitation of secreted BMP4 protein levels in 24-h conditioned medium. *n* = 3/group, mean ± SEM. **d** RT-qPCR analysis of the expression of canonical target genes in 231-HM cells with modified levels of BMP4 and/or SMAD4. *n* = 3/group, mean ± SEM. **e** Effect of enforced BMP4 expression and/or SMAD4 knockdown on the proliferation of cultured 231-HM cells. 500 cells were seeded on day 0 and proliferation was tracked for 5 days. *n* = 6/group, mean ± SEM. Statistical analysis was completed using the exponential growth curve equation function in Prism. ns, not significant. **f** Effect of enforced BMP4 expression and/or SMAD4 knockdown on colony formation of 231-HM cells. 60 cells were seeded on day 0 and colonies were counted on day 22. *n* = 3/group, mean ± SEM. For bar plots, statistical analysis was completed by Student’s t test. ns, not significant; *, *p* < 0.05; **, *p* < 0.01; ***, *p* < 0.001; ****, *p* < 0.0001. See also Fig. S1

SMAD4 is a well-known tumor suppressor gene, first identified in pancreatic cancer and called DPC4 [22]. While SMAD4 is normally expressed in breast cancer where we have shown that BMP4 has anti-metastatic activity [8], in colorectal and pancreatic cancers where a pro-tumor role of BMP4 is observed [23, 24], SMAD4 is mutated or lost in up to 40% of cases [25]. In the absence of SMAD4, BMP4 can induce non-canonical signalling, including MAPK pathways [26, 27], primarily through TGFβ-activated kinase 1 (TAK1) [28, 29]. We therefore proposed that the anti-metastatic activity of BMP4 could be mediated by SMAD4-dependent canonical signalling, and upon the loss of functional SMAD4, BMP4 could promote cancer progression through non-canonical signalling pathways (Fig. 1a).

To understand more comprehensively the tumor responses that follow the activation of canonical BMP4 signalling through SMAD4, and non-canonical BMP4 signalling in the absence of SMAD4, we have utilized preclinical models of spontaneous breast cancer metastasis to investigate the impact of SMAD4 on the ability of BMP4 to regulate tumor growth and metastasis. Furthermore, to reveal mechanisms by which BMP4 regulates tumor progression in the presence and absence of SMAD4, we have completed transcriptomic analyses of the canonical and non-canonical signalling pathways that are modulated by BMP4.

## Methods

### Cell culture and in vitro assays

MDA-MB-231-HM (231-HM) cells, a highly metastatic derivative of MDA-MB-231 cells [30], were kindly gifted by Prof. ZM Shao at the Fudan University Cancer Institute (Shanghai, China). MDA-MB-468 (468) and HEK293T cells were obtained from ATCC. 231-HM and 468 cells were authenticated by short tandem repeat (STR) profiling and cultured in Dulbecco’s modified eagle medium (DMEM, Thermo Fisher Scientific #11965126), supplemented with 10% fetal calf serum (FCS, Bovogen #SFBS) and penicillin/streptomycin (Thermo Fischer #15140122). Highly metastatic 4T1.2 mouse mammary cancer cells were derived from the 4T1 BALB/c mouse mammary cancer line [31], and cultured in alpha minimum essential medium Eagle (MEMα, Thermo Fisher Scientific #12561056), supplemented with 5% FCS and penicillin/streptomycin. Cells were maintained in a humidified incubator at 37 °C with 5% CO_2_, routinely screened for mycoplasma contamination (MycoAlert, Lonza #LT07), and used within six passages for experiments.

### Plasmid cloning and lentiviral transduction

For lentivirus-mediated enforced expression of TurboGFP, luciferase2 (Luc2), mCherry, BMP4, SMAD4, BMPR1a^Q233D^ (caBMPR1a) and MYO1F, coding sequences (CDS) were PCR amplified using primers containing restriction enzyme sites as specified in Table S1. The template for caBMPR1a, pLenti6-BMPR1A(Q233D)-V5 was a gift from Daniel Haber (Addgene plasmid #35638). PCR products and pLV lentiviral vectors, developed by Tobias Meyer (Addgene plasmid #85132, #85134 and #85139) [32], were digested using the corresponding restriction enzymes and subsequently ligated using the T4 DNA ligase (New England BioLabs #M0202). SMAD4 shRNA constructs were purchased from Dharmacon (now Horizon/PerkinElmer) with the shRNA sequences specified in Table S1. HEK293T cells were transfected with the lentiviral constructs along with the packaging constructs, pCMV-VSV-G and pCMV-dR8.2 (Addgene plasmid #8454 and #8455) [33]. Conditioned medium containing lentiviral particles were filtered and transferred onto target cells. Cells with stable expression of target genes or shRNA constructs were selected in medium containing the corresponding antibiotics (10 μg/mL puromycin, 800 μg/mL hygromycin or 1.2 mg/mL G418) for two weeks.

### CRISPR knockout

The Alt-R CRISPR/Cas9 system (IDT) was used for SMAD4 knockout in mouse 4T1.2 cells. In brief, two crRNA (SMAD4KO.aa, GAGTACGTTCACGACTTTGA, PAM = AGG; SMAD4KO.ab, ACAACCCGCTCATAGTGATA, PAM = TGG) targeting different regions of the *Smad4* exon were duplexed with ATTO550-labelled tracrRNA (IDT #1075927) to form two different guide RNAs (gRNAs). Each gRNA was complexed with the Cas9 nuclease to form a ribonucleoprotein (RNP) complex that was transfected into 4T1.2 cells using lipofectamine CRISPRMAX (Thermo Fisher Scientific #CMAX00001). Single cell clones of ATTO550^high^ RNP-transfected cells were expanded and screened by western blotting for SMAD4 deletion. To minimize the loss of heterogeneity compared to the parental cell line, we pooled five single cell clones generated using each of the two gRNAs in which SMAD4 loss at the protein level was confirmed. These pooled clones were used for subsequent experiments.

### Western blotting

Note that generic chemicals used in this study were purchased from Sigma-Aldrich or VWR unless specified otherwise. Cells or mechanically homogenized mouse tissues were lysed in cold lysis buffer (2% SDS and 50 mM Tris–HCl, pH 7 with 1 × Halt phosphatase/protease inhibitor cocktail, Thermo Fisher #78428) and incubated at 95 °C for 10 min. Fifteen to fifty micrograms of proteins in loading buffer (0.1 M DTT, 4% glycerol and 0.0004% bromophenol blue in lysis buffer) were loaded onto Bolt Bis–Tris mini protein gels (Thermo Fisher #NW0412C) and run at 200 V in MOPS buffer (Thermo Fisher #NP0001) for approximately 35 min. Proteins were transferred onto PVDF membranes (Merk Millipore #IPVH00010) using a Bolt mini blot module at 20 V for 75 min. Membranes were blocked with 5% skim milk in PBS for 1 h and incubated with primary antibodies (for concentrations, see Table S2) at 4 °C overnight or at room temperature for 2 h. Following incubation with HRP-conjugated secondary antibodies (BioRad #170–6515 or 170–6516) at room temperature for 1 h, protein bands were detected with the Western Lightning-Plus enhanced chemiluminescence (ECL) substrate (PerkinElmer #NEL103001EA). When stripping was required, membranes were washed with warm mild stripping buffer (1.5% glycine, 0.1% SDS and 1% Tween 20, pH 2.2) four times for 5 min each time, or incubated with harsh stripping buffer (2% SDS, 0.8% 2-mercaptoethanol and 62.5 mM Tris–HCl, pH 6.8) at 50 °C for 30 min, followed by blocking and antibody detection.

### BMP4 ELISA

In 6-well plates, 24-h conditioned medium from cells was recovered and centrifuged sequentially at 400 g and 2,000 g to remove cells and debris. The concentration of secreted BMP4 protein was determined using a human BMP4 DuoSet ELISA kit (R&D systems #DY314) and normalized to the protein concentration in each well.

### Quantitative reverse transcription PCR (RT-qPCR)

Total RNA from cells or tissues was isolated using Trizol (Thermo Fisher #15596026) according to the manufacturer’s instructions. Residual DNA was removed using the TURBO DNA-free kit (Thermo Fisher #AM1907). Single strand cDNA was synthesized using the ProtoScript II reverse transcriptase (New England Biolabs #M0368) with random pentadecamers. Gene expression was determined using the SYBR green real-time PCR master mix (Thermo Fisher Scientific #4385612) with target-specific forward and reverse primers (for details, see Table S3). Ribosomal protein S27a (*Rps27a*) or ribosomal protein L37a (*RPL37a*) was used as an internal control for mouse or human genes, respectively.

### In vitro proliferation and colony formation assays

In vitro proliferation rates of cells were determined with a sulforhodamine B (SRB) assay as described previously [34]. Cells (500 for 231-HM and 4T1.2 cells, and 1,000 for 468-GIL cells) were seeded into each well of 96-well plates in full medium and proliferation was tracked before cells reach confluency (5 days for 231-HM and 4T1.2 cells, and 11 days for 468-GIL cells). At each time point, cells were fixed in 3.3% trichloroacetic acid (TCA) at 4 °C overnight and stained with 0.057% SRB in 1% acetic acid at room temperature for 30 min. Plates were washed with 1% acetic acid and dried. SRB was dissolved in Tris base solution (10 mM, pH 10.5) and the optical density (OD) of the solution at 564 nm was determined.

For colony formation assays, 60 cells were seeded into each well of 6-well plates in full medium, followed by 12 or 22 days of incubation at 37 °C. The resulting colonies were fixed and stained with 0.1% crystal violet in 1:1 methanol:water at room temperature for 30 min. Colonies consisting of more than 50 cells were counted manually for each well.

### In vivo metastasis models

All animal experiments were approved by the Austin Health Animal Ethics Committee prior to commencement. Animals were housed in a clean and temperature-regulated facility with free access to food and water. One million TurboGFP- and luciferase-tagged human breast cancer cells (231-HM or 468-GIL variants) in 1:1 PBS:matrigel, or 100,000 mCherry-tagged mouse mammary cancer cells (4T1.2 variants) in PBS, were injected into the inguinal mammary fat pad of NOD *scid* gamma (NSG) mice or BALB/c mice, respectively, for the establishment of orthotopic tumors. Tumor volume was monitored by calliper measurements and calculated as ½(length × width^2^). Tumors were surgically resected when they reached 400 mm^3^ in volume. Luciferase-tagged metastatic lesions were visualized in the IVIS spectrum in vivo imaging system (PerkinElmer) following intraperitoneal injection of luciferin (3 mg/mouse in PBS). Mice were humanely euthanized 15 days after surgery for 231-HM and 4T1.2 models, 69 days after surgery for the 468 model, or when they developed signs of ill-health due to metastatic disease. Metastatic lesions in different organs were visualized ex vivo using the Maestro2 multispectral imaging system (CRi).

### Immunohistochemistry

Resected tumors or recovered organs at endpoint were fixed in neutral buffered formalin (10%) at room temperature for 24 h. Fixed tissues were dehydrated in increasingly concentrated ethanol (70% to 100%), incubated in xylene, and embedded in paraffin (Leica Biosystems #3960). Sections (4–8 μm) were rehydrated and subjected to heat-induced antigen retrieval either in an acidic buffer (0.05% Tween 20 and 10 mM citrate, pH 6) or in a basic buffer (1 mM EDTA, 0.05% Tween 20 and 10 mM Tris–HCl, pH 8). Following inactivation of endogenous hydrogen peroxidases in hydrogen peroxide (3% in PBST), sections were blocked in normal goat serum (3% in PBST) and incubated with primary antibodies at 4 °C overnight. Following incubation with biotin-conjugated secondary antibodies at room temperature for 1 h and signal amplification using HRP-conjugated avidin–biotin complexes (ABC, Vector #PK-4000), protein signals were detected using the 3,3'-diaminobenzidine (DAB, Dako #K3468) substrate. Sections were counterstained with haematoxylin, rinsed with Scott’s tap water, dehydrated in ethanol and xylene, and mounted in Entellan. Details of primary antibodies and the corresponding antigen retrieval methods can be found in Table S2.

### Metastatic burden analysis

Organs containing metastatic lesions were homogenized mechanically in lysis buffer (0.1 mg/mL proteinase K, 100 mM NaCl, 25 mM EDTA, 5% SDS and 10 mM Tris–HCl, pH 8) and incubated at 55 °C overnight. Excess protein was precipitated by incubation with 0.7 volumes of saturated NaCl solution (> 5 M) on ice for 30 min. Genomic DNA in the supernatant was extracted by phenol/chloroform/isoamyl alcohol (25:24:1, supplemented with 0.1% 8-hydroxyquinoline) with ethanol precipitation of DNA in the separated aqueous phase. Metastatic burden was assessed by qPCR quantitation of tumor cell-specific DNA (*TurboGFP* for human cancer cells or *mCherry* for mouse cancer cells), with *Rps27a* or *Vimentin* as the DNA quantity control, respectively, in extracted genomic DNA. Primers and probes used in this assay are specified in Table S3. Metastatic burden is calculated as 2 ^ -(CT_*TurboGFP*_ – CT_*Rps27a*_) × 10,000 for human xenografts, or as 2 ^ -(CT_*mCherry*_ – CT_*Vimentin*_) × 10,000 for mouse tumors.

### RNA sequencing and bioinformatics

231-HM tumors with or without the expression of BMP4 and the knockdown of SMAD4 were resected when they reached 400 mm^3^ in volume. Tumors were minced manually into fine pieces and further dissociated in collagenase type IV (1 mg/mL in DMEM) at 37 °C for 45 min with constant agitation. Cells were passed through a 70 μm strainer, resuspended in red blood cell lysis buffer (155 mM NH_4_Cl, 10 mM KHCO_3_ and 0.1 mM EDTA) and incubated at room temperature for 5 min. Lysis was stopped by adding 1 volume of PBS. Cells were passed through a 40 μm strainer before FACS recovery of TurboGFP-positive cells.

Total RNA from TurboGFP-positive 231-HM cells was extracted using the RNeasy micro kit with on-column DNase treatment. Samples from four different tumors with an RNA integrity number (RIN) above 9.9 and the highest RNA concentration, as determined by the Tapestation RNA ScreenTape (Agilent #5067–5576), were included for each tumor type in downstream analysis. cDNA library from purified mRNA was synthesized according to the TruSeq RNA sample preparation workflow (Illumina #RS-122–2001). The pooled library containing 16 indexed samples was sequenced in four lanes using the NextSeq 500/550 high output kit (75 cycles, Illumina #20024906) at the La Trobe Genomics Platform (Bundoora, Victoria, Australia). The quality of the sequencing output was checked with the FastQC quality control tool [35]. RNA sequencing data have been deposited at the NCBI Gene Expression Omnibus (GSE199628).

A general analysis workflow published by Law et al*.* [36] was followed. Sequence alignment and feature counting were completed in R using the Rsubread package [37] and the genome reference consortium human build 38 (hg38) assembly. Lowly expressed genes with counts per million (CPM) values lower than 0.5 (equivalent to approximately less than 10 counts) in more than 12 samples were removed. Gene expression was normalized and analysed using the edgeR [38] and limma [39] packages. Gene set testing was completed using clusterProfiler [40].

For gene correlation and patient outcome analyses, the Metabric dataset [41] containing matched mRNA expression profiles and clinical information of 1,904 patients was downloaded from cBioPortal. mRNA expression z-scores relative to all samples (log microarray) were used to represent gene expression levels in downstream analysis. Overall survival analysis was completed using the coxph function in the survival package based on average gene expression, which fits a Cox proportional hazards regression model [42].

### Statistical analysis

Unless specified, unpaired Student’s t test was performed to determine the statistical significance in experiments with two groups, and multiple comparison with one-way analysis of variance (ANOVA) was performed to determine the statistical significance in experiments with more than two groups. Unless specified, error bars in figures represent standard error of the mean (SEM).

## Results

### BMP4 promotes tumor growth in the absence of SMAD4 but suppresses metastasis independent of tumor intrinsic SMAD4 expression

To distinguish the consequences of SMAD4-dependent (canonical) and SMAD4-independent (non-canonical) signalling (Fig. 1a), we generated several tumor lines with modified levels of SMAD4 and/or BMP4. Using a highly metastatic variant of the triple-negative MDA-MB-231 human breast cancer line, MDA-MB-231-HM (231-HM) [30], we reduced SMAD4 expression using two short-hairpin RNAs (shRNAs), SMAD4sh4 and SMAD4sh5, that target different regions of the SMAD4 gene (Fig. 1b). Control cells were transduced with a non-silencing control shRNA (NonSil). The resulting cells were transduced with BMP4, leading to sustained BMP4 expression (Fig. 1b) and secretion (Fig. 1c), and activation of SMAD1/5/8 phosphorylation (Fig. 1b). In the presence of SMAD4, BMP4 expression resulted in a significant upregulation of known canonical target genes, including inhibitors of DNA binding 1–3 (*ID1, ID2, ID3*), *SMAD6* and *SMAD7* (Fig. 1d). However, when SMAD4 was reduced, the basal and/or BMP4-induced expression of these target genes was suppressed (Fig. 1d), indicative of reduced canonical signalling activity. Modifications of SMAD4 and/or BMP4 expression did not alter the proliferation (over 5 days) or colony-forming capacity (over 22 days) of 231-HM cells in vitro (Fig. 1e,f).

MAPK pathways have been reported by others to be activated in non-canonical signalling [26, 27] whereas PI3K/AKT signalling can be activated by BMP4 during development [43, 44]. We did not note any changes in the levels of XIAP, a putative upstream mediator of non-canonical signalling [45, 46], ERK1/2 phosphorylation, or p38 phosphorylation following deletion of SMAD4 (Fig. S1a-d). Interestingly, enforced expression of BMP4 resulted in a trend of increased AKT1 phosphorylation in both SMAD4-expressing and SMAD4-knockdown cells (Fig. S1a,e), consistent with previous reports of a similar effect by BMP2 in other cancers [47, 48].

**Fig. 2:**
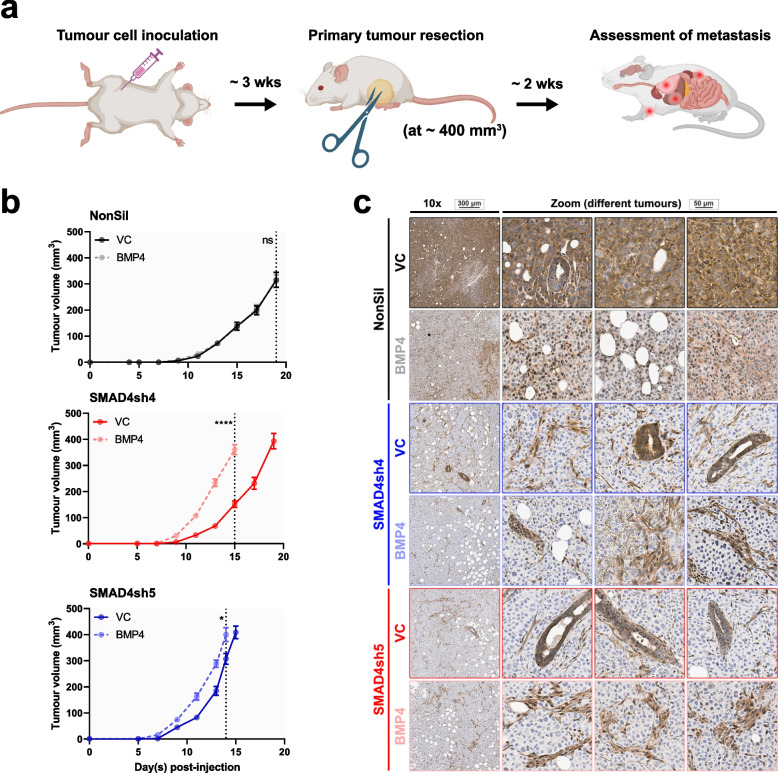
Non-canonical BMP4 signalling, but not canonical signalling, promotes tumor growth. **a** Timeframe for tumor growth and onset of metastatic disease of the 231-HM tumor model. Cells (1,000,000) were injected into the mammary glands of NSG mice. Tumor growth was tracked before resection until approximately 400 mm^3^. Metastatic burden was assessed 15 days after resection. Created with BioRender.com. **b** Effect of enforced BMP4 expression on SMAD4-expressing (top) and SMAD4-knockdown (middle and lower panels) 231-HM tumors. *n* = 15/group, mean ± SEM. **c** Immunohistochemical analysis of the levels of SMAD4 in resected 231-HM tumors, shown at both low and high magnification in different tumors

Orthotopic 231-HM tumors were established in the inguinal mammary gland of NOD *scid* gamma (NSG) mice (Fig. 2a). In the presence of SMAD4, enforced expression of BMP4 did not change the rate of tumor growth up to the day of resection (Fig. 2b). In contrast, BMP4 accelerated the growth of tumors with reduced SMAD4 expression (Fig. 2b), implicating a tumor growth-promoting role of non-canonical signalling. Modified expression of SMAD4 at the protein level was confirmed by immunohistochemistry in these primary tumors (Fig. 2c). As expected, enforced expression of BMP4 led to the nuclear translocation of SMAD4. In SMAD4-knockdown tumors, SMAD4 was absent from the tumor cells but persisted in the mouse stromal cells (Fig. 2c).

**Fig. 3:**
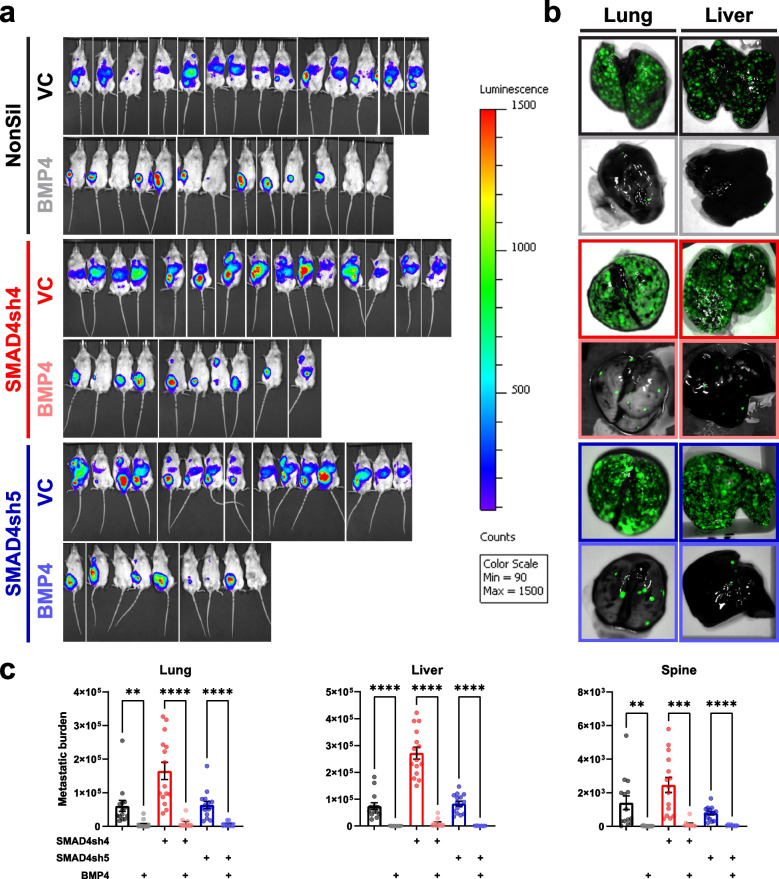
BMP4 suppresses metastasis independent of tumor-intrinsic expression of SMAD4. **a** Visualisation of luciferase-tagged metastatic lesions in mice using the IVIS Spectrum imaging system. **b** Representative images of TurboGFP-tagged metastatic lesions in the lungs and livers visualized ex vivo using the Maestro imaging system. **c** Metastatic burden in the lungs (left panel), livers (middle panel) and spines (right panel) at endpoint, as quantitated by determining the levels of tumor-specific *TurboGfp* genomic DNA in each organ. n ≥ 9/group, mean ± SEM. Statistical analysis was completed by Student’s t test. ns, not significant; *, *p* < 0.05; **, *p* < 0.01; ***, *p* < 0.001; ****, *p* < 0.0001. See also Fig. S2

Primary 231-HM tumors were resected at 400–500 mm^3^ in volume and at similar weights, but at different times after tumor cell inoculation due to differences in tumor growth rates (Fig. S2a). In mice bearing control tumors (with control vector and NonSil hairpin), extensive metastasis to the lungs, liver and bones was observed in the absence of BMP4, as evidenced by in vivo imaging of tumor-specific luciferase activity (Fig. 3a), ex vivo imaging of TurboGFP-tagged metastatic lesions (Fig. 3b) and qPCR quantification of tumor-specific *TurboGfp* DNA in different organs (Fig. 3c). Enforced expression of BMP4 in the NonSil tumors led to a systemic reduction in metastasis (Fig. 3a-c), as we have shown previously in other tumor models [7, 8], and was associated with a reduction in tumor-induced splenomegaly (Fig. S2b). The luciferase signal that is still evident in the mice expressing BMP4 is due to regrowth of the inguinal mammary gland primary tumor after resection. Surprisingly, even though BMP4 promoted the growth of SMAD4-knockdown primary tumors (Fig. 2b), the anti-metastatic impact of BMP4 was retained despite this loss of SMAD4 activity (Fig. 3a-c).

To confirm this unexpected observation of BMP4-induced suppression of metastasis even when SMAD4 levels are low, we utilized a second model, the human triple-negative MDA-MB-468 breast cancer line that lacks SMAD4 due to a homozygous deletion [49]. TurboGFP- and luciferase-tagged cells (468-GIL) were transduced with BMP4 and a low titre of SMAD4 vector (Fig. S3a), since we discovered that 468-GIL cells transduced with a high titre of SMAD4 were not viable in culture, potentially due to restored activity of anti-proliferative TGFβ signalling [50]. Enforced expression of BMP4 did not alter the proliferation (11 days) of SMAD4-null or SMAD4-expressing cells in vitro (Fig. S3b). The induction of canonical target genes by BMP4 was restored upon enforced expression of SMAD4 (Fig. S3c). Consistent with results from the 231-HM model, BMP4 promoted the growth of SMAD4-null 468-GIL tumors in NSG mice (Fig. S3d). This enhanced tumor growth persisted when low levels of SMAD4 expression were enforced, however, in the presence of BMP4, the time required for 468-GIL tumors expressing SMAD4 to reach 400 mm^3^ was notably longer (60 days) compared to that for the SMAD4-null tumors (40 days) (Fig. S3d). BMP4 significantly suppressed lung metastasis from SMAD4-null 468-GIL tumors with a trend towards reduced lung metastasis from SMAD4-expressing 468-GIL tumors (Fig. S3e).

We next asked if a similar outcome would be observed when assessing SMAD4-depleted tumors in immunocompetent mice. To achieve this, we modified the highly metastatic 4T1.2 triple-negative mammary tumor line with CRISPR/Cas9 mediated knockout of SMAD4 using two different guide RNA complexes (SMAD4KO.aa and SMAD4KO.ab), followed by enforced expression of BMP4 in the SMAD4 knockout cells (Fig. S4a). Each of these tumor lines was pooled with five single cell clones generated using the corresponding gRNA. This was due to our need to minimize the loss of heterogeneity given its importance in the metastatic potential of tumor lines [51]. Although we acknowledge that the pooled tumor lines are potentially less heterogeneous compared to the parental line, the approach of using a bulk population of edited cells would not have been appropriate, as the knockout efficiency (approximately 22% for SMAD4KO.aa and 50% for SMAD4KO.ab) was not sufficient to minimize or eliminate SMAD4 activity. Similar to the two human lines, the upregulation of *Id1*, *Id2* and *Smad6* by BMP4 was suppressed upon loss of SMAD4, while that of *Id3* and *Smad7* was retained to a limited extent (Fig. S4b). Altered expression of SMAD4 or BMP4 did not impact proliferation (5 days) or colony forming capacity (12 days) of 4T1.2 cells in vitro (Fig. S4c,d). The pro-tumor growth effect of BMP4 noted in 231-HM SMAD4-knockdown and 468-GIL SMAD4-null tumors was not observed in SMAD4-knockout 4T1.2 tumors (Fig. S4e), possibly due to differences in the tumor models and/or due to an active host immune response in the BALB/c mice [7]. However, consistent with results from the other two models, the anti-metastatic effect of BMP4 was retained in 4T1.2 tumor-bearing mice despite total loss of SMAD4 (Fig. S4f).

### The suppression of metastasis is dependent on SMAD4 activity when BMP signalling is restricted to the tumor cells

**Fig. 4:**
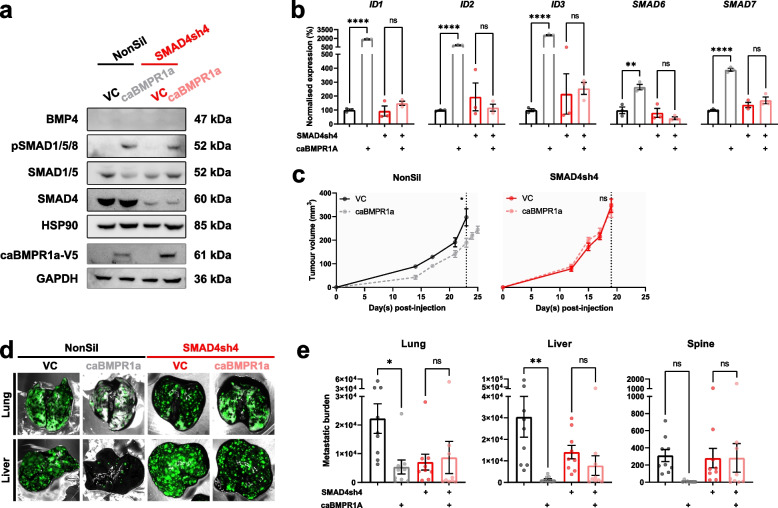
Tumor cell-specific activation of BMP signalling suppresses metastasis in a SMAD4-dependent manner. **a** Western blotting validation of enforced expression of a constitutively active type I BMP receptor (caBMPR1a) in SMAD4 expressing and SMAD4 depleted 231-HM cells. **b** RT-qPCR analysis of the expression of canonical target genes in 231-HM cells with enforced expression of caBMPR1a and/or knockdown of SMAD4. *n* = 3/group, mean ± SEM. **c** Effect of enforced expression of caBMPR1a on the growth of SMAD4-expressing (left panel) or SMAD4-knockdown (right panel) 231-HM tumors. *n* = 9/group, mean ± SEM. **d** TurboGFP-tagged metastatic lesions in the lungs and livers were visualized using the Maestro imaging system at endpoint. **e** Metastatic burden in the lungs (left), liver (middle) and spine (right) at endpoint. *n* = 9/group, mean ± SEM. Statistical analysis was completed by Student’s t test. ns, not significant; *, *p* < 0.05; **, *p* < 0.01; ***, *p* < 0.001; ****, *p* < 0.0001

BMP4 is a secreted protein that is known to act on stromal cells [7]. In BMP4-expressing 231-HM tumors that lacked tumor-intrinsic SMAD4 expression, SMAD4 was still present in stromal cells (Fig. 2c), and canonical BMP4 signalling in the stroma was still likely to be functional. To understand if *tumor cell intrinsic* BMP signalling requires SMAD4 for suppression of metastasis and to eliminate the paracrine influence of secreted BMP4 on stromal cells, we generated tumor cells with a constitutively active Type Ia BMP receptor, BMPR1a^Q233D^ (caBMPR1a), thereby removing the requirement for BMP4 to initiate the signalling. The resulting cells were then transduced with the SMAD4-knockdown shRNA construct (Fig. 4a). Enforced expression of caBMPR1a led to sustained phosphorylation of SMAD1/5/8 (Fig. 4a) and the induction of canonical target genes (Fig. 4b). Upon reduction of SMAD4 expression, caBMPR1a-induced upregulation of canonical target genes was largely abrogated (Fig. 4b).

Orthotopic 231-HM tumors with caBMPR1a were established in NSG mice. There was a modest reduction in primary tumor growth in 231-HM-caBMPR1a-expressing cells in the presence but not in the absence of SMAD4 (Fig. 4c). Consistent with the anti-metastatic activity of BMP4, constitutively active BMPR1a in SMAD4 positive tumors displayed a significant reduction in metastasis to lung, liver and spine (Fig. 4d,e), however, in the absence of SMAD4, the suppression of metastasis was largely abrogated (Fig. 4d,e), indicating that SMAD4 activity is required for inhibition of metastasis following tumor cell-specific BMP signalling.

### Transcriptomic analysis reveals mechanisms of increased tumor growth induced by non-canonical BMP4 signalling

Since BMP4 promoted the growth of tumors with low or no SMAD4 activity, the use of BMP4 to activate signalling as a therapy in tumors with weak expression of SMAD4 might lead to adverse effects despite its anti-metastatic activity. Non-canonical signalling pathways involved in this promotion of tumor growth may explain the contradictory effect of BMP4 in regulating tumor progression that has been reported by others [23, 52–54]. To address this, we next investigated the mechanisms by which non-canonical BMP4 signalling promotes tumor growth.

**Fig. 5:**
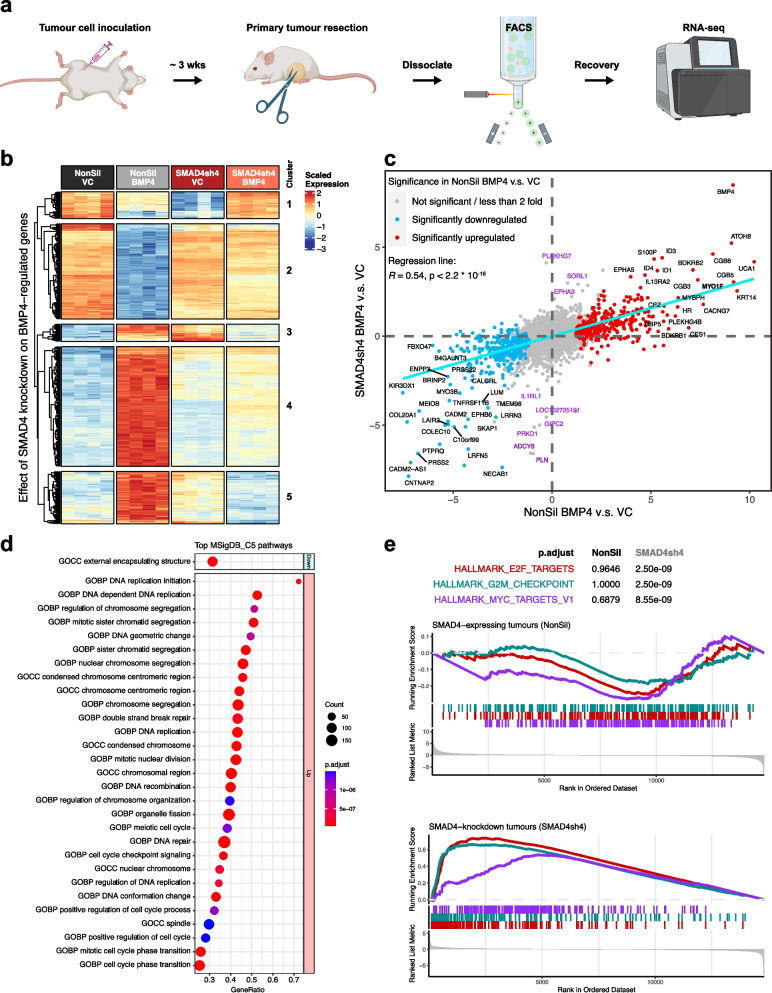
Transcriptomic analysis reveals that non-canonical BMP4 signalling drives DNA replication and cell cycle progression. **a** Workflow of recovery of 231-HM cells from primary tumors for RNA sequencing. Compared to Fig. 2a, resected tumors were dissociated, and TurboGFP-tagged tumor cells were recovered by FACS for downstream RNA sequencing analysis. Created with BioRender.com. **b** Heatmap of differentially upregulated and downregulated genes in 231-HM tumor cells with modified expression of BMP4 and/or SMAD4. **c** XY plot of the correlation between the logFC induced by enforced BMP4 expression in SMAD4-expressing (x axis) and in SMAD4-knockdown (y axis) tumors. Genes that were uniquely upregulated or downregulated by BMP4 in the absence of SMAD4 are coloured in purple. MYO1F that was investigated in Fig. S6 is highlighted in bold. **d** Gene set enrichment analysis of gene ontology pathways regulated by BMP4 specifically in SMAD4-knockdown tumors compared to SMAD4-expressing tumors. Top 30 pathways by significance are shown. **e** Enrichment plots illustrating BMP4-induced gene expression changes in the E2F_targets, the G2M_checkpoint and the MYC_targets hallmark pathways in SMAD4-expressing (upper panel) and SMAD4-knockdown (lower panel) tumors. See also Fig. S5

Orthotopic 231-HM tumors with or without modified expression of SMAD4 and/or BMP4 were established in NSG mice. TurboGFP-tagged cancer cells were recovered from resected primary tumors via fluorescence-activated cell sorting (FACS) and subjected to RNA sequencing analysis (Fig. 5a).

At the level of individual genes, the top 500 genes (by significance) that were identified to be differentially regulated by BMP4 in the presence or absence of SMAD4 are visualized as a heatmap in Fig. 5b. We validated these results by completing RT-qPCR analysis of some of the most significantly regulated genes (Fig. S5).

Five clusters of genes were observed: genes downregulated by BMP4 in SMAD4-expressing tumors but upregulated in SMAD4-knockdown tumors (Fig. 5b, cluster 1); genes downregulated or upregulated by BMP4 in SMAD4-expressing tumors, but remain relatively unaltered in SMAD4-knockdown tumors (Fig. 5b, clusters 2 and 4, respectively); and genes upregulated by BMP4 in SMAD4-expressing tumors but were downregulated in SMAD4-knockdown tumors (Fig. 5b, clusters 3 and 5). A correlation plot of BMP4-induced gene expression changes in SMAD4-expressing and in SMAD4-knockdown tumors is shown in Fig. 5c.

Although a large proportion of genes showed similar patterns of BMP4-induced expression changes in the presence *vs* in the absence of SMAD4 (Fig. 5c, cyan line) indicating a significant correlation of gene expression changes, a subset of genes was differentially regulated only in SMAD4-knockdown tumors (Fig. 5c, purple).

To identify genes that were specifically regulated by canonical or non-canonical signalling (i.e., in the presence or in the absence of SMAD4, respectively), bioinformatic analysis of gene expression changes was completed with the following comparison: 

$$\text{log}\left(\frac{{Expression}_{SMAD4sh4.BMP4}}{{Expression}_{SMAD4sh4.VC}}\right)-\text{log}\left(\frac{{Expression}_{NonSil.BMP4}}{{Expression}_{NonSil.VC}}\right)$$

At the level of signal transduction in the absence of SMAD4, gene set enrichment analysis revealed that BMP4 preferentially upregulated DNA replication, chromosomal regulation and cell cycle-related ontology gene sets (Fig. 5d). Indeed, whilst enforced BMP4 expression in SMAD4-expressing tumors did not lead to significant changes in the E2F targets, G2M checkpoint or MYC targets pathways in the Hallmark gene set (Fig. 5e, upper panel), genes associated with these pathways were significantly upregulated by BMP4 in the absence of SMAD4 (Fig. 5e, lower panel).

### BMP4-induced MYO1F upregulation suppresses metastasis

Next, we sought to identify potential mediators of the anti-metastatic effect. While ID1 was the most significantly upregulated in SMAD4-expressing tumors (Table S4), it is a well-known target of BMP4 and has been implicated in the promotion of metastasis in different cancer types [16, 55, 56]. The next most significantly upregulated gene was MYO1F. Tumor cell expression of MYO1F was increased to a greater degree by BMP4 in the presence of SMAD4 than in its absence (Figs. 5c and S5). At the protein level, we detected elevated MYO1F in BMP4 expressing primary tumors, but only in the presence of SMAD4 (Fig. S6a).

MYO1F is a member of the class 1 myosin family of motor proteins, involved mainly in cell contractility, using energy from ATP to drive molecular trafficking, cell adhesion and migration [57]. It is reported to be highly expressed in neutrophils but is also expressed in cancer cells [58, 59], co-localising with actin and membrane phospholipids [60]. We have not found any prior studies that directly investigate the effect of MYO1F on metastasis, and therefore decided to explore the possible metastasis function of this gene in breast cancer.

To determine if MYO1F was causally involved in suppression of metastasis, we enforced expression of MYO1F in parental 231-HM cells (Fig. S6b). The presence of MYO1F did not alter primary tumor growth (Fig. S6c) but substantially suppressed metastasis to lung, liver and spine (Fig. S6d,e). Thus, BMP4 induced MYO1F is acting as a metastasis suppressor.

### Clinical relevance of BMP4-induced canonical and non-canonical signalling

To evaluate the clinical relevance of BMP4-induced canonical and non-canonical signalling pathways, we examined the expression levels and prognostic values of genes that are associated with these pathways in the Metabric breast cancer patient dataset [41].

**Fig. 6:**
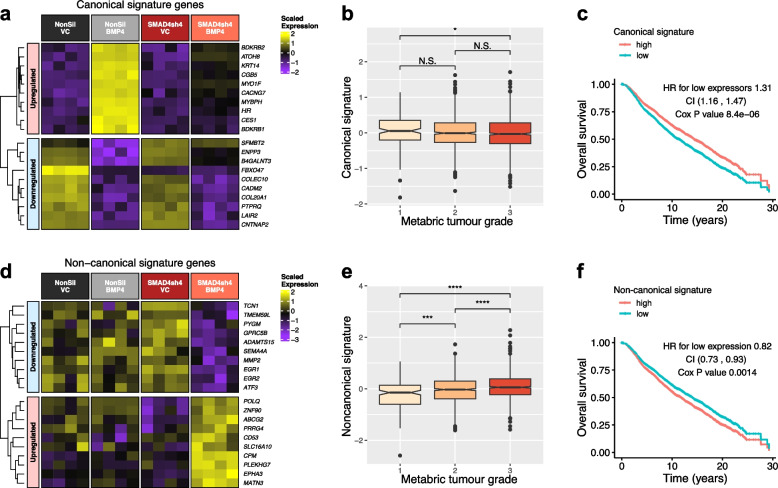
Prognostic value of canonical and non-canonical BMP4 signalling in breast cancer patients included in the Metabric dataset. **a** Expression of genes included in the canonical signature in 231-HM tumors with modified expression of BMP4 and/or SMAD4. **b** Canonical signature score was calculated based on the upregulated genes minus the down-regulated genes from (**a**). Average signature scores for tumors of different grades in the Metabric dataset are visualized. **c** Correlation between canonical signature score and overall survival in breast cancer patients. **d** Expression of genes included in the non-canonical signature in 231-HM tumors with modified expression of BMP4 and/or SMAD4. **e** Non-canonical signature score was calculated based on the upregulated genes minus the down-regulated genes from (**d**). Average signature scores for tumors of different grades in the Metabric dataset are visualized. **f** Correlation between non-canonical signature score and overall survival in breast cancer patients. Statistical analysis was completed by the stat_compare_means function in R for (**b**) and (**e**). N.S., not significant; *, *p* < 0.05; **, *p* < 0.01; ***, *p* < 0.001; ****, *p* < 0.0001. Statistical analysis was completed by the coxph function in R for (**c**) and (**f**). HR, hazard ratio; CI, confidence interval; Cox, Cox proportional-hazards model

For canonical, SMAD4-dependent BMP4 signalling, we generated a signature based on the most upregulated genes minus the most down-regulated genes that were also available in the Metabric dataset (Fig. 6a) and assessed expression and prognostic value in breast cancer patients. The canonical signature was decreased in patients with Grade 3 tumors (Fig. 6b) while high levels of the signature were prognostic for improved overall survival (Fig. 6c).

For non-canonical, SMAD4-independent BMP4 signalling, marker genes for this pathway have not previously been reported. We identified genes that were specifically regulated by BMP4 in the absence of SMAD4 based on our RNA sequencing data with the following criteria: (1) genes significantly regulated by BMP4 in SMAD4-knockdown tumors; (2) genes not significantly regulated by BMP4 in SMAD4-expressing tumors; (3) genes not significantly affected by SMAD4 knockdown alone; and (4) genes where the difference in gene expression changes induced by BMP4 in SMAD4-knockdown tumors versus those in SMAD4-expressing tumors was significant. A few genes (ten in total) that were not available in the Metabric dataset were omitted. The expression levels of the top genes that satisfied these criteria in 231-HM tumors with modified levels of BMP4 and/or SMAD4 are visualized in a heatmap in Fig. 6d.

A signature score for non-canonical signalling was generated based on these genes using the same method for Fig. 6b,c. In patients, high-grade breast tumors showed elevated levels of non-canonical signalling-associated genes (Fig. 6e). Furthermore, high levels of non-canonical signalling-associated genes predicted worse overall survival of patients (Fig. 6f).

## Discussion

In previous studies, we have reported that BMP4 potently suppresses metastasis in preclinical mouse models where breast cancer cells retained SMAD4 activity to transduce canonical signalling. We have also reported that high levels of BMP4 expression are associated with favourable patient outcome, especially when assessed in combination with high levels of canonical BMP4 target genes such as SMAD7 [7, 8]. In this study, we have sought to understand the reason for the conflicting reports of the impact of BMP4 on progression of different types of cancer [61], especially in cancers of the gastrointestinal tract [16] or the pancreas [24] where cancer promoting activities of BMP4 were identified.

We hypothesized that the activity of SMAD4 mediates the anti-metastatic activity of BMP4, and that when SMAD4 is lost, non-canonical BMP4 signalling promotes tumor growth and metastasis [19]. In agreement with our previous reports [7, 8] but contradicting a report by another group where a trend towards accelerated experimental metastasis in BMP4-treated mice was observed [13], in 231-HM and 4T1.2 tumors that expressed SMAD4, enforced BMP4 expression did not alter tumor growth kinetics (Figs. 2b and S4e). Consistent with our hypothesis, when SMAD4 activity was reduced by two different shRNA constructs, enforced BMP4 accelerated tumor growth in the 231-HM tumor model (Fig. 2b). A similar response was observed in mice bearing SMAD4-null MDA-MB-468 tumors (Fig. S3d). These findings indicate that a BMP4-activating therapy has the potential to enhance tumor growth in patients bearing tumors with low or no SMAD4 activity, consistent with previous reports in colorectal cancer [62] and pancreatic cancer [63].

As anticipated, enforced BMP4 expression led to a profound inhibition of spontaneous metastasis to multiple organs in mice bearing tumors that expressed SMAD4 (Figs. 3, S3e and S4f). To our surprise and contrary to our hypothesis, in all three models, the anti-metastatic effect of BMP4 was retained when SMAD4 activity was either reduced or abrogated (Figs. 3, S3e and S4f). It has been reported that BMP signalling in stromal cells of the tumor microenvironment, such as fibroblasts, could influence the metastatic propensity of mammary tumors [64, 65]. Indeed, we have shown previously that BMP4 can suppress the number and activity of myeloid derived suppressor cells in the tumor microenvironment [7]. To avoid paracrine signalling by BMP4, we enforced the expression of a constitutively active BMP receptor, caBMPR1a, in the 231-HM tumor cells. While caBMPR1a expression led to a significant reduction in metastasis, this anti-metastatic response was lost in tumors with reduced SMAD4 activity (Fig. 4d,e). We conclude from these studies that the presence of BMP4 is required to suppress metastasis via paracrine mechanisms, even in tumors with loss of SMAD4 activity and that the reason why BMP4 can promote metastasis in gastrointestinal cancers still remains elusive.

For breast cancer, these results provide evidence supporting the selective use of BMP receptor agonists to inhibit breast cancer metastasis. Indeed, a recent study by Ren et al. [66] has found that when used in combination with MAPK kinase (MEK) inhibitors, a small molecule activator of BMP signalling, tacrolimus (also called FK506), could effectively suppress metastasis in the MDA-MB-231 tumor model. Since tacrolimus is used to suppress immune rejection of tissue transplants, it may not be suitable for cancer therapy.

To identify causes of the adverse effects of non-canonical BMP4 signalling, we completed RNA sequencing analysis of 231-HM cells recovered from an in vivo tumor microenvironment. By comparing BMP4-induced transcriptomic changes in SMAD4-expressing tumors and those in SMAD4-knockdown tumors, we identified genes and pathways that were regulated by BMP4 specifically in the absence of SMAD4, and that may contribute to non-canonical signalling (Fig. 5c,d). Some non-canonical BMP4 target genes identified in this study, such as EPHA3 and SORL1, have been implicated in the progression of breast cancer [67, 68]. At the pathway level, BMP4 promoted DNA replication and cell cycle progression through SMAD4-independent signalling (Fig. 5d), whereas this effect of BMP4 was not observed in SMAD4-expressing tumors (Fig. 5e). These findings are consistent with a previous report where cell cycle progression of hepatocellular carcinoma cells was accelerated by BMP4 treatment independent of SMAD4, potentially via the induction of CDK1 and cyclin B1 [69].

One of the top upregulated genes in both SMAD4 expressing and SMAD4 knockdown tumors, MYO1F, was analysed further and found to be a metastasis suppressor (Fig. S6). Thus, BMP4 appears to suppress metastasis, at least in part, by upregulation of MYO1F. To the best of our knowledge, MYO1F has not previously been linked to tumor progression [70].

Finally, we constructed a signature comprising genes that were specifically regulated by BMP4 in the absence of SMAD4 and found that this signature is increasingly represented in high-grade breast tumors (Fig. 6e) and predicts significantly worse overall survival (Fig. 6f) in the Metabric patient dataset. Activation of these non-canonical pathways by BMP4 may explain the association between high levels of BMP4 expression and chemotherapy resistance in non-small cell lung cancer [71] and in gastric cancer [72].

In conclusion, our study demonstrates that the anti-metastatic effect of BMP4 is mediated by SMAD4-dependent signalling that is transduced either in the cancer cells or in the stromal cells. The tumor promoting effect of BMP4 is potentially mediated by SMAD4-independent non-canonical signalling that leads to accelerated DNA replication and cell cycle progression, which needs to be considered if patients with SMAD4-low/null tumors were to receive therapies that activate BMP signalling.

### Limitations of the study

We assessed the requirement for tumor cell expression of SMAD4 in mediating responses to BMP4, demonstrating that BMP4 is required for suppression of metastasis, regardless of the SMAD4 status of the tumor cells. Since BMP4 is a secreted cytokine, this leaves open the possibility that BMP4 acts in a paracrine manner to trigger changes in the tumor microenvironment to suppress metastasis. We have shown in an earlier study that BMP4 can suppress the activity of myeloid derived suppressor cells, but this is unlikely to be the only mechanism since BMP4 also suppresses metastasis in highly immunocompromized mice. In addition, we still cannot explain why BMP4 acts to drive tumor progression in some other cancer types, since SMAD4 status appears to not be the major factor. We will continue to investigate this topic in future studies.

### Supplementary Information

**Additional file 1: Supplementary Figure 1.** Additional information for Fig. 1. (a) Western blotting analysis of non-canonical signalling pathways in 231-HM cells with modified expression of BMP4 and/or SMAD4. (b-e) Densitometry analysis of expression levels of XIAP (b), and phosphorylation levels of p-ERK1/2 (c), p-p38 (d) and p-AKT1 (e). *n *= 3/group, mean ± SEM. Statistical analysis was completed by Student’s t-test. ns, not significant. *P* values < 0.3 are shown.

**Additional file 2: Supplementary Figure 2.** Additional information for Fig. 3. (a) Weights of 231-HM tumors at resection. Tumors were resected at the same volume (approximately 400 mm^3^) on different days. n ≥ 9/group, mean ± SEM. (b) Spleen weights of mice bearing 231-HM tumors at endpoint, as an indicator of the overall metastatic burden. n ≥ 9/group, mean ± SEM. Statistical analysis was completed by Student’s t test. ns, not significant; *, *p* <0.05; **, *p* <0.01; ***, *p* <0.001; ****, *p* <0.0001.

**Additional file 3: Supplementary Figure 3.** Modification of the MDA-MB-468 (468-GIL) breast cancer model to investigate the effect of canonical and non-canonical BMP4 signalling. (a) Western blotting validation of enforced expression of BMP4, SMAD1/5/8 signalling and low levels of SMAD4 in 468-GIL cells. (b) *In vitro* proliferation of cells expressing BMP4 and/or SMAD4. 1,000 cells were seeded on day 0 and proliferation was tracked for 11 days. *n* = 6/group, mean ± SEM. Statistical analysis was completed using the exponential growth curve equation function in Prism. ns, not significant. (c) RT-qPCR analysis of the expression of canonical target genes in 468-GIL cells with modified levels of BMP4 and/or SMAD4. *n* = 3/group, mean ± SEM. (d) Effect of BMP4 on the growth of SMAD4-null (left panel) or SMAD4-expressing (right panel) 468-GIL tumors. Cells (1,000,000) were injected into the mammary glands of NSG mice. *n* = 9/group, mean ± SEM. (e) Metastatic burden in the lungs of 468-GIL tumor-bearing mice at endpoint (69 days after resection). *n* = 9/group, mean ± SEM. For bar plots, statistical analysis was completed by Student’s t test. ns, not significant; *, *p* <0.05; **, *p* <0.01; ***, *p *<0.001; ****, *p* <0.0001.

**Additional file 4: Supplementary Figure 4.** Effect of modifying BMP4 and/or SMAD4 levels on the growth and metastatic response of 4T1.2 tumors. (a) Western blotting validation of enforced BMP4 expression, SMAD1/5/8 signalling and SMAD4 knockout in 4T1.2 cells. (b) RT-qPCR analysis of the expression of canonical target genes in 4T1.2 cells with modified levels of BMP4 and/or SMAD4. *n* = 3/group, mean ± SEM. (c) Effect of enforced BMP4 expression and/or SMAD4 knockout on the proliferation of cultured 4T1.2 cells. 500 cells were seeded on day 0 and proliferation was tracked for 5 days. *n* = 6/group, mean ± SEM. Statistical analysis was completed using the exponential growth curve equation function in Prism. ns, not significant. (d) Effect of enforced BMP4 expression and SMAD4 knockout on colony formation of cultured 4T1.2 cells. 60 cells were seeded on day 0 and colonies were counted on day 12. *n* = 3/group, mean ± SEM. (e) Effect of enforced BMP4 expression on SMAD4-expressing and SMAD4-knockout tumors. Cells (100,000) were injected into the mammary glands of BALB/c mice. *n* = 12/group. (f) Metastatic burden in the lungs was quantitated by determining the levels of tumor-specific *mCherry* genomic DNA in each organ at endpoint (15 days after resection). n ≥ 10/group. For bar plots, statistical analysis was completed by Student’s t test. ns, not significant; *, *p* <0.05; **, *p* <0.01; ***, *p* <0.001; ****, *p* <0.0001.

**Additional file 5: Supplementary Figure 5.** Confirmation of RNA sequencing results by RT-PCR. RT-qPCR analysis of BMP4-regulated genes that were identified in the RNA sequencing analysis. RNA extracted from *in vitro* cultured 231-HM cells. *n* = 3/group, mean ± SEM. Statistical analysis was completed by Student’s t test. ns, not significant; *, *p* <0.05; **, *p* <0.01; ***, *p* <0.001; ****, *p* <0.0001.

**Additional file 6: Supplementary Figure 6.** The role of MYO1F in regulation of metastasis. (a) Western blotting validation of MYO1F upregulation by BMP4 in SMAD4-expressing 231-HM tumors. (b) Validation of stable exogenous expression of MYO1F in 231-HM parental cells. (c) Effect of enforced expression of MYO1F on the growth of 231-HM tumors. Cells (1,000,000) were injected into the mammary glands of NSG mice. *n* = 6/group, mean ± SEM. (d) TurboGFP-tagged metastatic lesions in the lungs and liver were visualized using the Maestro imaging system at endpoint (15 days after resection). *n* = 6/group, mean ± SEM. (e) Metastatic burden in the lungs, liver and spine at endpoint. *n* = 6/group, mean ± SEM. Statistical analysis was completed by Student’s t test in (c) and (e). N.S., not significant; *, *p* <0.05; **, *p* <0.01; ***, *p *<0.001; ****, *p* <0.0001.

**Additional file 7: Supplementary table 1.** Cloning primers and relevant shRNA sequences used in this study.

**Additional file 8: Supplementary table 2.** Western blotting / IHC antibodies used in this study.

**Additional file 9: Supplementary table 3.** Primers and probes for (RT-)qPCR analysis in this study.

**Additional file 10: Supplementary table 4.** Top 100 differentially regulated genes by BMP4 in SMAD4-expressing 231-HM tumors (by significance).

## Data Availability

Sequence data that support the findings of this study have been deposited at the NCBI Gene Expression Omnibus (GSE199628). The secure token was intended for the reviewers only. Upon publication, the dataset will be made available by our contacting the admin of NCBI GEO (with link, DOI or PMID to the published article).

## Abbreviations

ABC: Avidin biotin complex
ANOVA: Analysis of variance
ATCC: American Type Culture Collection
CDS: Coding sequence
CPM: Counts per million
CRISPR: Clustered regularly interspersed short palindromic repeats
crRNA: CRISPR RNA
CT: Threshold cycle
DAB: 3,3'-Diaminobenzidine
DMEM: Dulbecco’s modified eagle’s medium
DTT: Dithiothreitol
ECL: Enhance chemiluminescence
EDTA: Ethylenediamine tetraacetic acid
ELISA: Enzyme linked immunosorbent assay
EMT: Epithelial to mesenchymal transition
FCS: Fetal calf serum
G418: Gentamycin
gRNA: Guide RNA
HRP: Horseradish peroxidase
MDSC: Myeloid derived suppressor cells
MEMalpha: Minimal essential medium alpha
MOPS: 3-(N-morpholino)propanesulfonic acid
NonSil: Non-silencing control shRNA
NSG: Non-obese diabetic *scid* gamma
PBS: Phosphate buffered saline
PBST: PBS-Tween20
PCR: Polymerase chain reaction
PVDF: Polyvinyl
RIN: RNA integrity number
RNP: Ribonucleoprotein
RT-qPCR: Quantitative reverse transcriptase PCR
SDS: Sodium dodecyl sulphate
SRB: Sulforhodamine B
SEM: Standard error of the mean
TCA: Trichloroacetic acid
