# Supplementary figures and images for: Loss of tumor-derived SMAD4 enhances primary tumor growth but not metastasis following BMP4 signalling
Source: Cell Commun Signal. 2024 Apr 30;22:248. doi: 10.1186/s12964-024-01559-0 (PMC11060976; doi:10.1186/s12964-024-01559-0)

**(separate experiment)**

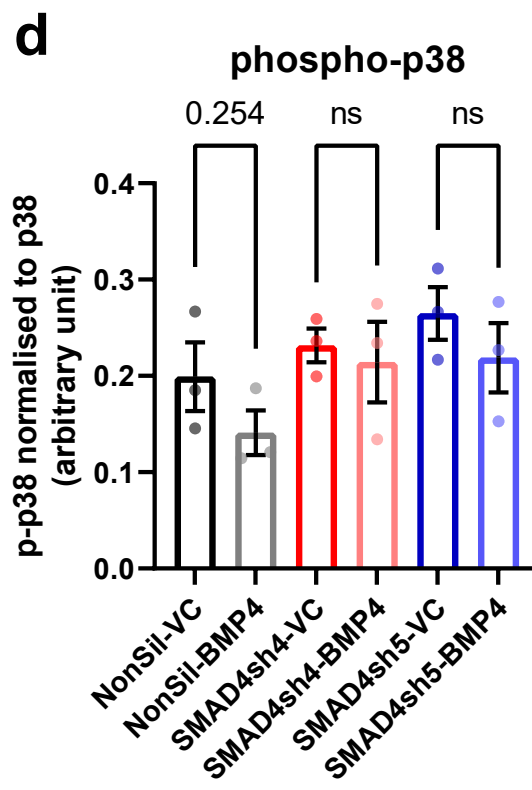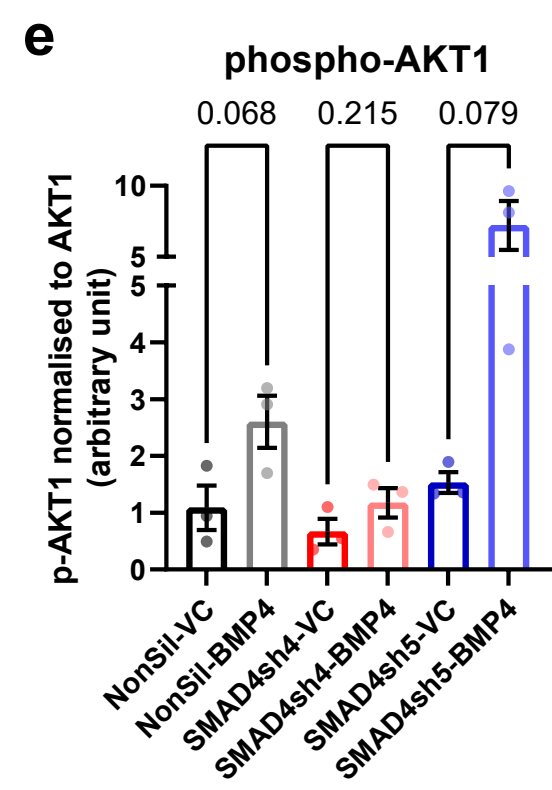

Supplement: Supplementary file 1 — Additional file 1: Supplementary Figure 1. Additional information for Fig. 1. (a) Western blotting analysis of non-canonical signalling pathways in 231-HM cells with modified expression of BMP4 and/or SMAD4. (b-e) Densitometry analysis of expression levels of XIAP (b), and phosphorylation levels of p-ERK1/2 (c), p-p38 (d) and p-AKT1 (e). n = 3/group, mean ± SEM. Statistical analysis was completed by Student’s t-test. ns, not significant. P values < 0.3 are shown. [file 12964_2024_1559_MOESM1_ESM.pdf]

Tumour weight (g)  
at resection

SMAD4sh4  
SMAD4sh5  
BMP4

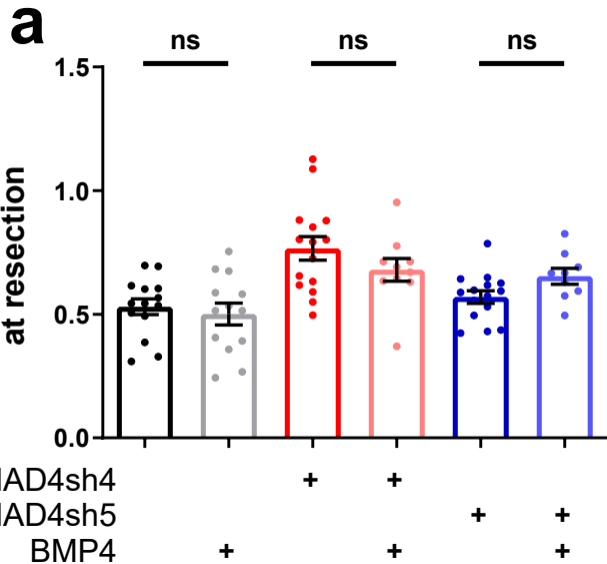

Spleen weight (g)  
at endpoint

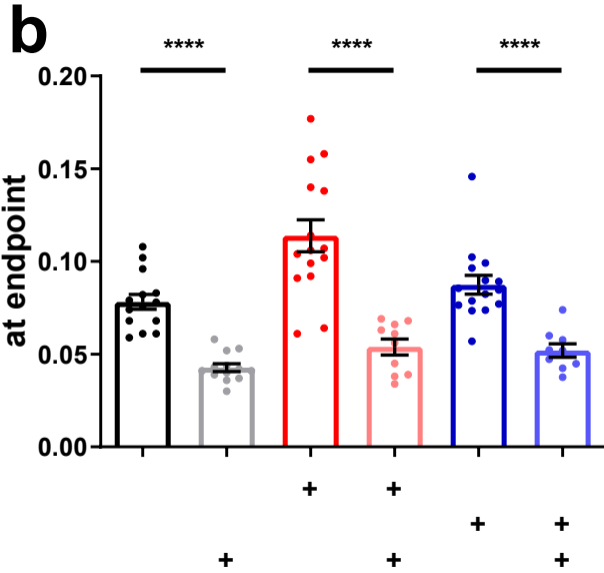

Supplement: Supplementary file 2 — Additional file 2: Supplementary Figure 2. Additional information for Fig. 3. (a) Weights of 231-HM tumors at resection. Tumors were resected at the same volume (approximately 400 mm3) on different days. n ≥ 9/group, mean ± SEM. (b) Spleen weights of mice bearing 231-HM tumors at endpoint, as an indicator of the overall metastatic burden. n ≥ 9/group, mean ± SEM. Statistical analysis was completed by Student’s t test. ns, not significant; *, p <0.05; **, p <0.01; ***, p <0.001; ****, p <0.0001. [file 12964_2024_1559_MOESM2_ESM.pdf]

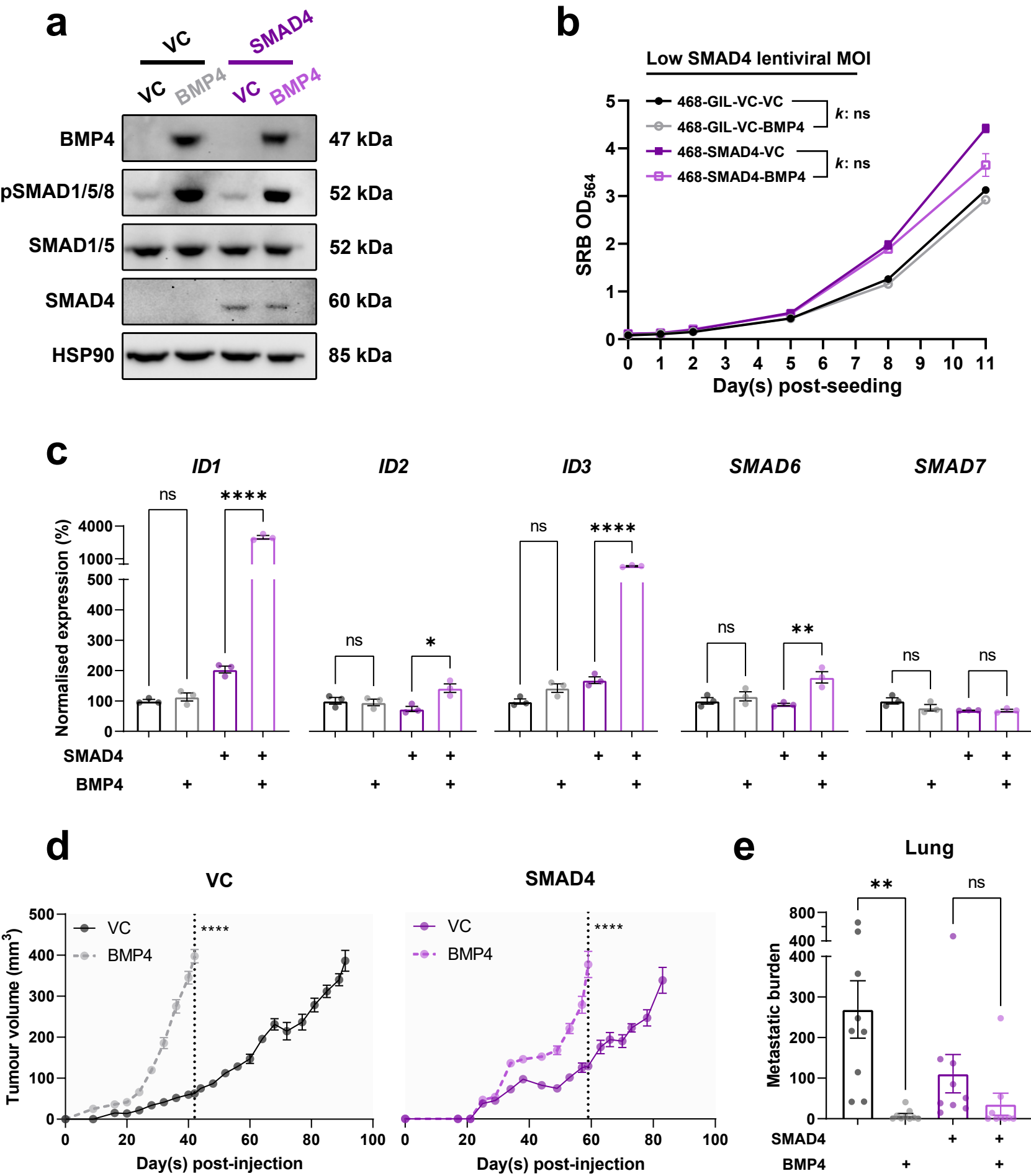

Supplement: Supplementary file 3 — Additional file 3: Supplementary Figure 3. Modification of the MDA-MB-468 (468-GIL) breast cancer model to investigate the effect of canonical and non-canonical BMP4 signalling. (a) Western blotting validation of enforced expression of BMP4, SMAD1/5/8 signalling and low levels of SMAD4 in 468-GIL cells. (b) In vitro proliferation of cells expressing BMP4 and/or SMAD4. 1,000 cells were seeded on day 0 and proliferation was tracked for 11 days. n = 6/group, mean ± SEM. Statistical analysis was completed using the exponential growth curve equation function in Prism. ns, not significant. (c) RT-qPCR analysis of the expression of canonical target genes in 468-GIL cells with modified levels of BMP4 and/or SMAD4. n = 3/group, mean ± SEM. (d) Effect of BMP4 on the growth of SMAD4-null (left panel) or SMAD4-expressing (right panel) 468-GIL tumors. Cells (1,000,000) were injected into the mammary glands of NSG mice. n = 9/group, mean ± SEM. (e) Metastatic burden in the lungs of 468-GIL tumor-bearing mice at endpoint (69 days after resection). n = 9/group, mean ± SEM. For bar plots, statistical analysis was completed by Student’s t test. ns, not significant; *, p <0.05; **, p <0.01; ***, p <0.001; ****, p <0.0001. [file 12964_2024_1559_MOESM3_ESM.pdf]

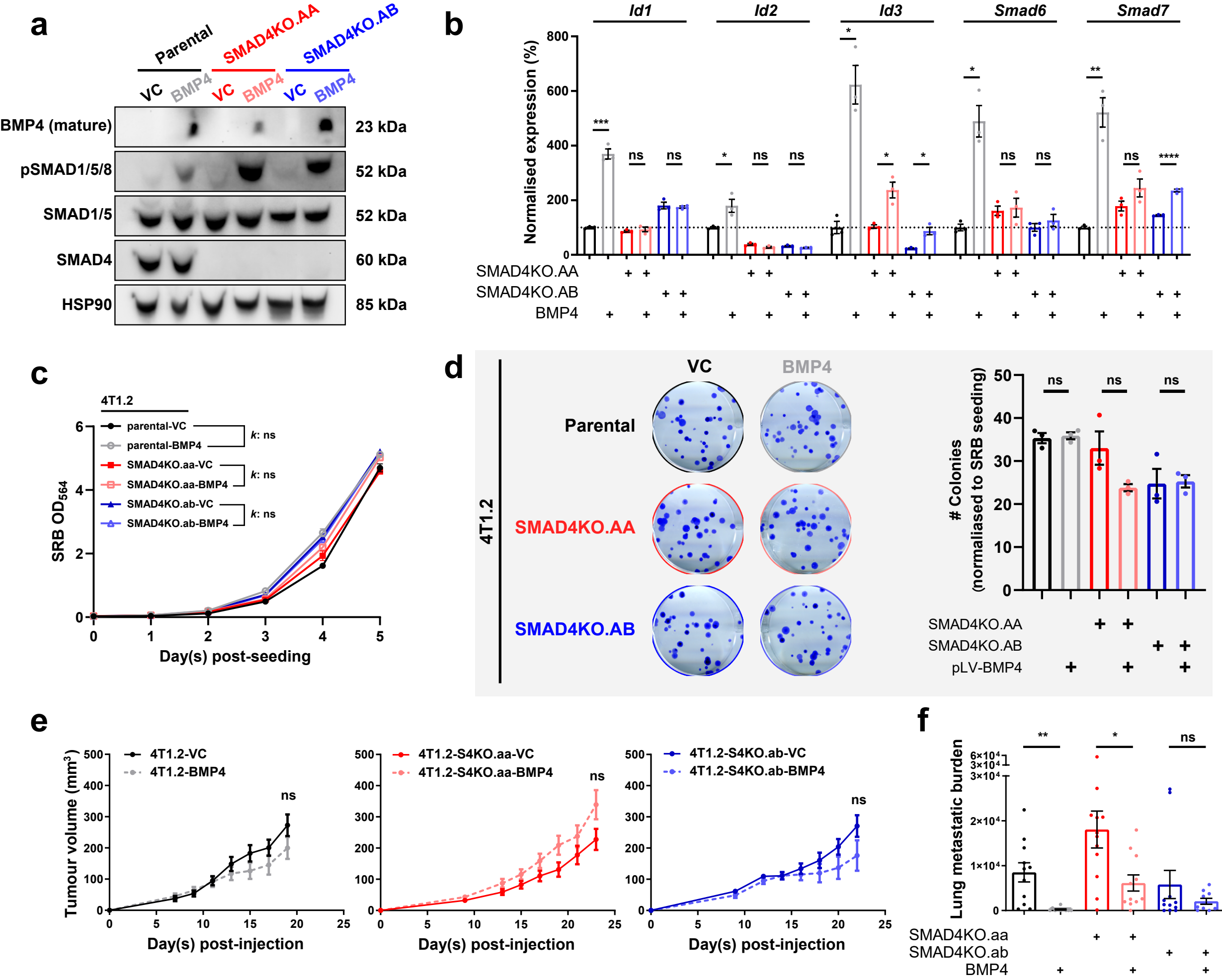

Supplement: Supplementary file 4 — Additional file 4: Supplementary Figure 4. Effect of modifying BMP4 and/or SMAD4 levels on the growth and metastatic response of 4T1.2 tumors. (a) Western blotting validation of enforced BMP4 expression, SMAD1/5/8 signalling and SMAD4 knockout in 4T1.2 cells. (b) RT-qPCR analysis of the expression of canonical target genes in 4T1.2 cells with modified levels of BMP4 and/or SMAD4. n = 3/group, mean ± SEM. (c) Effect of enforced BMP4 expression and/or SMAD4 knockout on the proliferation of cultured 4T1.2 cells. 500 cells were seeded on day 0 and proliferation was tracked for 5 days. n = 6/group, mean ± SEM. Statistical analysis was completed using the exponential growth curve equation function in Prism. ns, not significant. (d) Effect of enforced BMP4 expression and SMAD4 knockout on colony formation of cultured 4T1.2 cells. 60 cells were seeded on day 0 and colonies were counted on day 12. n = 3/group, mean ± SEM. (e) Effect of enforced BMP4 expression on SMAD4-expressing and SMAD4-knockout tumors. Cells (100,000) were injected into the mammary glands of BALB/c mice. n = 12/group. (f) Metastatic burden in the lungs was quantitated by determining the levels of tumor-specific mCherry genomic DNA in each organ at endpoint (15 days after resection). n ≥ 10/group. For bar plots, statistical analysis was completed by Student’s t test. ns, not significant; *, p <0.05; **, p <0.01; ***, p <0.001; ****, p <0.0001. [file 12964_2024_1559_MOESM4_ESM.pdf]

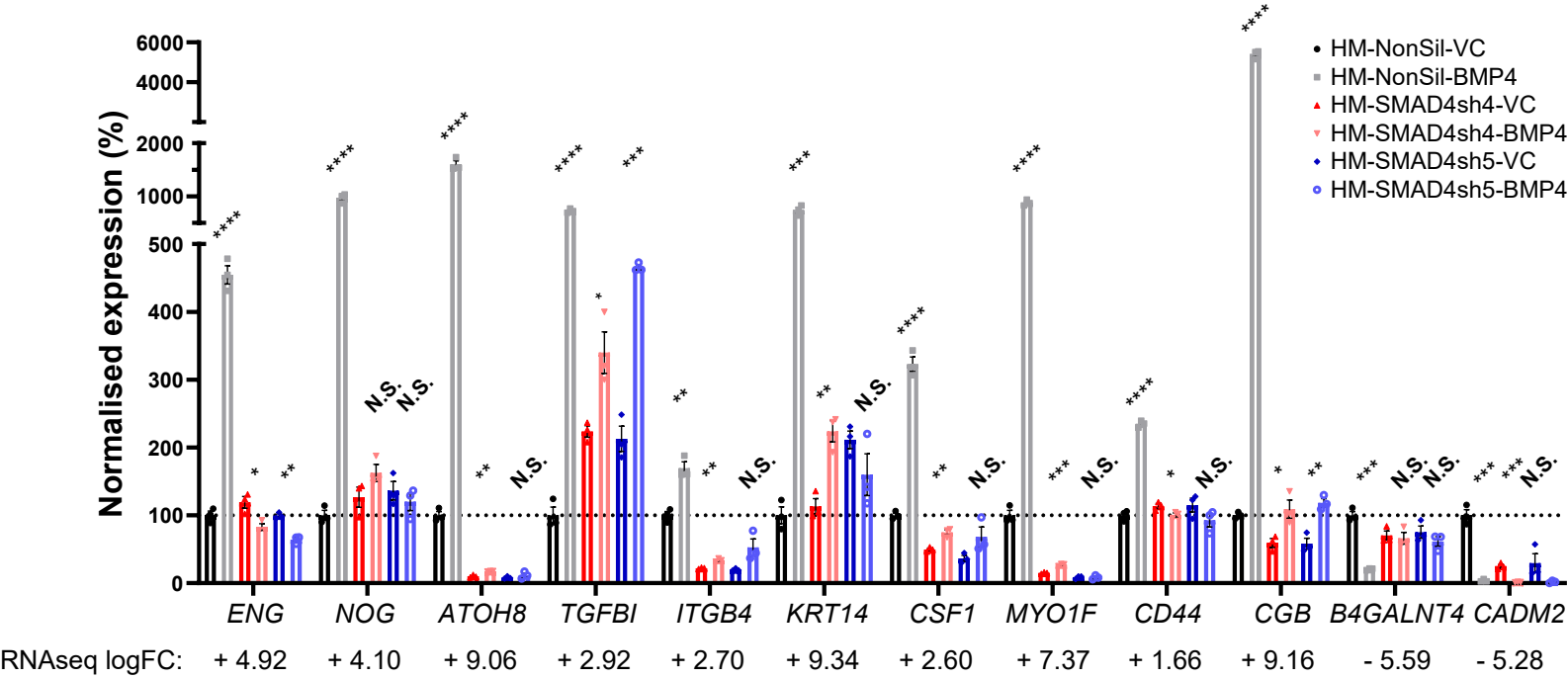

Supplement: Supplementary file 5 — Additional file 5: Supplementary Figure 5. Confirmation of RNA sequencing results by RT-PCR. RT-qPCR analysis of BMP4-regulated genes that were identified in the RNA sequencing analysis. RNA extracted from in vitro cultured 231-HM cells. n = 3/group, mean ± SEM. Statistical analysis was completed by Student’s t test. ns, not significant; *, p <0.05; **, p <0.01; ***, p <0.001; ****, p <0.0001. [file 12964_2024_1559_MOESM5_ESM.pdf]

# Supplementary figure 6

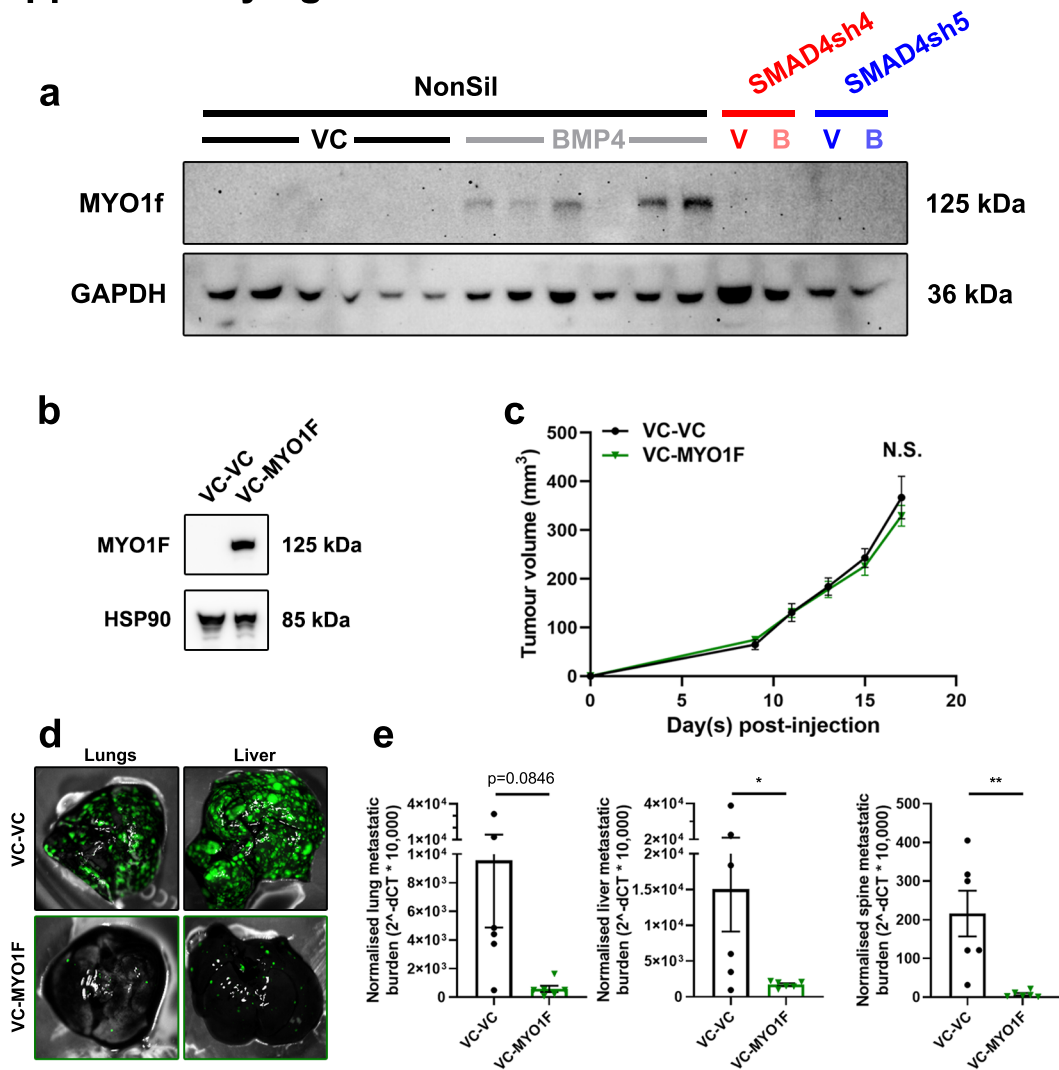

Supplement: Supplementary file 6 — Additional file 6: Supplementary Figure 6. The role of MYO1F in regulation of metastasis. (a) Western blotting validation of MYO1F upregulation by BMP4 in SMAD4-expressing 231-HM tumors. (b) Validation of stable exogenous expression of MYO1F in 231-HM parental cells. (c) Effect of enforced expression of MYO1F on the growth of 231-HM tumors. Cells (1,000,000) were injected into the mammary glands of NSG mice. n = 6/group, mean ± SEM. (d) TurboGFP-tagged metastatic lesions in the lungs and liver were visualized using the Maestro imaging system at endpoint (15 days after resection). n = 6/group, mean ± SEM. (e) Metastatic burden in the lungs, liver and spine at endpoint. n = 6/group, mean ± SEM. Statistical analysis was completed by Student’s t test in (c) and (e). N.S., not significant; *, p <0.05; **, p <0.01; ***, p <0.001; ****, p <0.0001. [file 12964_2024_1559_MOESM6_ESM.pdf]
