# Supplementary material for: Loss of tumor-derived SMAD4 enhances primary tumor growth but not metastasis following BMP4 signalling
Source: Cell Commun Signal. 2024 Apr 30;22:248. doi: 10.1186/s12964-024-01559-0 (PMC11060976; doi:10.1186/s12964-024-01559-0)
Supplement: Supplementary file 10 — Additional file 10: Supplementary table 4. Top 100 differentially regulated genes by BMP4 in SMAD4-expressing 231-HM tumors (by significance). [file 12964_2024_1559_MOESM10_ESM.docx]

**Table S4. Top 100 differentially regulated genes by BMP4 in SMAD4-expressing 231-HM tumours (by significance).**

| ***GeneID*** | ***Symbol*** | ***type_of_gene*** | ***logFC*** | ***AveExpr*** | ***t*** | ***P.Value*** | ***adj.P.Val*** |
| --- | --- | --- | --- | --- | --- | --- | --- |
| 3397 | ID1 | protein-coding | 5.32 | 5.97 | 49.23 | 3.41E-23 | 2.54E-19 |
| 5167 | ENPP1 | protein-coding | -4.10 | 7.98 | -37.73 | 7.84E-21 | 3.90E-17 |
| 4542 | MYO1F | protein-coding | 7.37 | 3.12 | 35.71 | 2.41E-20 | 8.98E-17 |
| 2022 | ENG | protein-coding | 4.92 | 5.23 | 35.06 | 3.48E-20 | 9.99E-17 |
| 9241 | NOG | protein-coding | 4.10 | 4.42 | 34.82 | 4.02E-20 | 9.99E-17 |
| 3399 | ID3 | protein-coding | 5.56 | 4.10 | 33.32 | 9.81E-20 | 2.09E-16 |
| 5330 | PLCB2 | protein-coding | 4.04 | 5.17 | 30.55 | 5.70E-19 | 1.06E-15 |
| 23136 | EPB41L3 | protein-coding | 4.17 | 2.92 | 26.54 | 9.67E-18 | 1.60E-14 |
| 9590 | AKAP12 | protein-coding | -2.89 | 8.62 | -26.35 | 1.12E-17 | 1.67E-14 |
| 3691 | ITGB4 | protein-coding | 2.70 | 7.39 | 25.42 | 2.31E-17 | 3.13E-14 |
| 23164 | MPRIP | protein-coding | 2.16 | 9.11 | 24.66 | 4.24E-17 | 5.27E-14 |
| 7045 | TGFBI | protein-coding | 2.92 | 8.91 | 23.47 | 1.14E-16 | 1.29E-13 |
| 1435 | CSF1 | protein-coding | 2.60 | 5.99 | 23.39 | 1.21E-16 | 1.29E-13 |
| 158135 | TTLL11 | protein-coding | 2.62 | 6.59 | 23.15 | 1.50E-16 | 1.49E-13 |
| 5362 | PLXNA2 | protein-coding | 2.18 | 7.63 | 23.00 | 1.69E-16 | 1.58E-13 |
| 4091 | SMAD6 | protein-coding | 4.60 | 2.66 | 22.71 | 2.19E-16 | 1.92E-13 |
| 283358 | B4GALNT3 | protein-coding | -5.59 | 4.91 | -22.29 | 3.19E-16 | 2.64E-13 |
| 54843 | SYTL2 | protein-coding | -2.60 | 6.71 | -22.08 | 3.82E-16 | 2.98E-13 |
| 124976 | SPNS2 | protein-coding | -3.60 | 6.93 | -22.03 | 4.00E-16 | 2.98E-13 |
| 1795 | DOCK3 | protein-coding | 2.78 | 5.24 | 21.96 | 4.26E-16 | 3.02E-13 |
| 222663 | SCUBE3 | protein-coding | 4.06 | 3.40 | 21.67 | 5.56E-16 | 3.77E-13 |
| 1509 | CTSD | protein-coding | 2.15 | 9.81 | 21.60 | 5.91E-16 | 3.83E-13 |
| 26999 | CYFIP2 | protein-coding | -2.43 | 5.68 | -21.24 | 8.24E-16 | 5.12E-13 |
| 5047 | PAEP | protein-coding | -3.33 | 8.52 | -21.09 | 9.54E-16 | 5.69E-13 |
| 1293 | COL6A3 | protein-coding | 4.28 | 3.81 | 20.97 | 1.06E-15 | 6.09E-13 |
| 84985 | FAM83A | protein-coding | 3.49 | 3.00 | 20.12 | 2.41E-15 | 1.33E-12 |
| 9945 | GFPT2 | protein-coding | -1.98 | 7.32 | -19.61 | 3.97E-15 | 2.11E-12 |
| 100131897 | INSYN2B | protein-coding | 4.52 | 2.29 | 19.56 | 4.21E-15 | 2.16E-12 |
| 257019 | FRMD3 | protein-coding | 2.94 | 5.45 | 19.49 | 4.48E-15 | 2.23E-12 |
| 388610 | TRNP1 | protein-coding | 2.66 | 4.99 | 19.36 | 5.12E-15 | 2.46E-12 |
| 4093 | SMAD9 | protein-coding | 3.84 | 3.31 | 19.13 | 6.46E-15 | 3.01E-12 |
| 3339 | HSPG2 | protein-coding | 2.31 | 6.72 | 18.46 | 1.30E-14 | 5.88E-12 |
| 3887 | KRT81 | protein-coding | 3.61 | 2.58 | 18.35 | 1.46E-14 | 6.40E-12 |
| 1140 | CHRNB1 | protein-coding | -2.41 | 6.23 | -18.08 | 1.95E-14 | 8.32E-12 |
| 652995 | UCA1 | ncRNA | 10.20 | -1.32 | 17.88 | 2.47E-14 | 1.02E-11 |
| 8111 | GPR68 | protein-coding | 3.52 | 3.14 | 17.70 | 2.95E-14 | 1.18E-11 |
| 55504 | TNFRSF19 | protein-coding | -3.37 | 4.39 | -17.68 | 3.01E-14 | 1.18E-11 |
| 79789 | CLMN | protein-coding | -4.30 | 3.59 | -17.48 | 3.75E-14 | 1.43E-11 |
| 478 | ATP1A3 | protein-coding | 4.34 | 3.45 | 17.46 | 3.86E-14 | 1.44E-11 |
| 10974 | ADIRF | protein-coding | 2.97 | 4.89 | 17.04 | 6.14E-14 | 2.23E-11 |
| 26047 | CNTNAP2 | protein-coding | -7.26 | -1.44 | -16.76 | 8.57E-14 | 2.97E-11 |
| 5420 | PODXL | protein-coding | 2.17 | 8.26 | 16.75 | 8.57E-14 | 2.97E-11 |
| 54899 | PXK | protein-coding | 2.29 | 4.95 | 16.35 | 1.37E-13 | 4.64E-11 |
| 163071 | ZNF114 | protein-coding | 3.19 | 3.65 | 16.32 | 1.42E-13 | 4.71E-11 |
| 23129 | PLXND1 | protein-coding | 2.12 | 7.47 | 16.27 | 1.50E-13 | 4.85E-11 |
| 9124 | PDLIM1 | protein-coding | -2.14 | 7.09 | -16.05 | 1.96E-13 | 6.20E-11 |
| 51363 | CHST15 | protein-coding | -2.47 | 5.73 | -15.89 | 2.37E-13 | 7.36E-11 |
| 3899 | AFF3 | protein-coding | 3.71 | 3.04 | 15.87 | 2.42E-13 | 7.38E-11 |
| 84913 | ATOH8 | protein-coding | 9.06 | -0.69 | 15.69 | 3.06E-13 | 9.13E-11 |
| 5570 | PKIB | protein-coding | -2.72 | 3.39 | -15.63 | 3.25E-13 | 9.49E-11 |
| 22836 | RHOBTB3 | protein-coding | 2.04 | 8.19 | 15.50 | 3.83E-13 | 1.10E-10 |
| 4648 | MYO7B | protein-coding | 3.07 | 3.77 | 15.26 | 5.15E-13 | 1.45E-10 |
| 100874038 | CADM2-AS1 | ncRNA | -7.16 | -1.84 | -15.15 | 5.90E-13 | 1.63E-10 |
| 83478 | ARHGAP24 | protein-coding | 3.91 | 1.08 | 15.06 | 6.60E-13 | 1.79E-10 |
| 4052 | LTBP1 | protein-coding | -2.53 | 7.30 | -14.99 | 7.24E-13 | 1.93E-10 |
| 4608 | MYBPH | protein-coding | 6.34 | -1.22 | 14.80 | 9.20E-13 | 2.41E-10 |
| 55806 | HR | protein-coding | 6.23 | -1.08 | 14.66 | 1.11E-12 | 2.83E-10 |
| 3861 | KRT14 | protein-coding | 9.34 | -0.93 | 14.66 | 1.12E-12 | 2.83E-10 |
| 7851 | MALL | protein-coding | 2.94 | 2.55 | 14.63 | 1.15E-12 | 2.85E-10 |
| 494188 | FBXO47 | protein-coding | -6.05 | -3.76 | -14.54 | 1.28E-12 | 3.12E-10 |
| 2840 | GPR17 | protein-coding | 3.44 | 2.91 | 14.46 | 1.42E-12 | 3.42E-10 |
| 57605 | PITPNM2 | protein-coding | 1.95 | 5.86 | 14.35 | 1.64E-12 | 3.88E-10 |
| 11033 | ADAP1 | protein-coding | 3.45 | 2.01 | 14.31 | 1.74E-12 | 4.05E-10 |
| 221416 | C6orf223 | ncRNA | -4.26 | 3.64 | -14.13 | 2.19E-12 | 5.02E-10 |
| 2239 | GPC4 | protein-coding | -2.92 | 3.66 | -14.12 | 2.24E-12 | 5.06E-10 |
| 5651 | TMPRSS15 | protein-coding | 2.54 | 4.11 | 13.70 | 3.94E-12 | 8.76E-10 |
| 5157 | PDGFRL | protein-coding | -2.93 | 2.69 | -13.65 | 4.20E-12 | 9.21E-10 |
| 219699 | UNC5B | protein-coding | 3.35 | 2.21 | 13.54 | 4.88E-12 | 1.05E-09 |
| 10365 | KLF2 | protein-coding | 2.51 | 5.59 | 13.51 | 5.09E-12 | 1.08E-09 |
| 153478 | PLEKHG4B | protein-coding | 5.82 | -0.54 | 13.43 | 5.66E-12 | 1.19E-09 |
| 2624 | GATA2 | protein-coding | 3.28 | 1.86 | 13.41 | 5.85E-12 | 1.21E-09 |
| 57509 | MTUS1 | protein-coding | -2.78 | 3.82 | -13.21 | 7.74E-12 | 1.58E-09 |
| 140688 | NOL4L | protein-coding | 1.92 | 5.72 | 13.02 | 1.01E-11 | 2.03E-09 |
| 1066 | CES1 | protein-coding | 6.90 | -2.85 | 12.91 | 1.19E-11 | 2.36E-09 |
| 6678 | SPARC | protein-coding | 3.45 | 4.19 | 12.85 | 1.29E-11 | 2.54E-09 |
| 3169 | FOXA1 | protein-coding | -2.30 | 4.26 | -12.76 | 1.47E-11 | 2.85E-09 |
| 59284 | CACNG7 | protein-coding | 7.64 | -1.44 | 12.74 | 1.51E-11 | 2.89E-09 |
| 83959 | SLC4A11 | protein-coding | -2.15 | 7.62 | -12.72 | 1.56E-11 | 2.92E-09 |
| 57642 | COL20A1 | protein-coding | -7.34 | -0.45 | -12.72 | 1.57E-11 | 2.92E-09 |
| 93659 | CGB5 | protein-coding | 9.16 | -1.85 | 12.59 | 1.91E-11 | 3.52E-09 |
| 9469 | CHST3 | protein-coding | 1.99 | 5.55 | 12.49 | 2.18E-11 | 3.96E-09 |
| 2289 | FKBP5 | protein-coding | -2.06 | 6.30 | -12.38 | 2.55E-11 | 4.58E-09 |
| 112937 | GLB1L3 | protein-coding | 3.97 | 1.54 | 12.37 | 2.58E-11 | 4.58E-09 |
| 126014 | OSCAR | protein-coding | 2.60 | 4.40 | 12.36 | 2.62E-11 | 4.60E-09 |
| 90011 | KIR3DX1 | pseudo | -7.56 | 0.41 | -12.29 | 2.96E-11 | 5.13E-09 |
| 4092 | SMAD7 | protein-coding | 2.17 | 4.80 | 12.18 | 3.46E-11 | 5.93E-09 |
| 4188 | MDFI | protein-coding | 4.87 | 1.64 | 12.14 | 3.66E-11 | 6.20E-09 |
| 4192 | MDK | protein-coding | -1.98 | 6.50 | -11.98 | 4.63E-11 | 7.76E-09 |
| 126353 | MISP | protein-coding | -2.41 | 2.55 | -11.91 | 5.17E-11 | 8.57E-09 |
| 79603 | CERS4 | protein-coding | -3.64 | 1.81 | -11.80 | 6.09E-11 | 9.99E-09 |
| 4804 | NGFR | protein-coding | 4.72 | -0.19 | 11.76 | 6.57E-11 | 1.06E-08 |
| 94115 | CGB8 | protein-coding | 8.12 | -1.62 | 11.74 | 6.77E-11 | 1.09E-08 |
| 1191 | CLU | protein-coding | -1.85 | 6.79 | -11.68 | 7.34E-11 | 1.15E-08 |
| 335 | APOA1 | protein-coding | 4.68 | -0.39 | 11.68 | 7.35E-11 | 1.15E-08 |
| 7108 | TM7SF2 | protein-coding | -2.30 | 5.36 | -11.64 | 7.80E-11 | 1.21E-08 |
| 10461 | MERTK | protein-coding | -1.73 | 6.33 | -11.61 | 8.22E-11 | 1.25E-08 |
| 101059948 | LOC101059948 | ncRNA | 3.64 | 0.85 | 11.61 | 8.24E-11 | 1.25E-08 |
| 3398 | ID2 | protein-coding | 3.45 | 2.89 | 11.58 | 8.54E-11 | 1.29E-08 |
| 6693 | SPN | protein-coding | 4.25 | 0.34 | 11.53 | 9.28E-11 | 1.38E-08 |
| 57194 | ATP10A | protein-coding | 3.65 | 1.31 | 11.49 | 9.89E-11 | 1.46E-08 |
